# Novel haptens and monoclonal antibodies with subnanomolar affinity for a classical analytical target, ochratoxin A

**DOI:** 10.1038/s41598-018-28138-x

**Published:** 2018-06-27

**Authors:** Daniel López-Puertollano, Josep V. Mercader, Consuelo Agulló, Antonio Abad-Somovilla, Antonio Abad-Fuentes

**Affiliations:** 1 Department of Organic Chemistry, University of Valencia, Doctor Moliner 50, 46100 Burjassot, Valencia Spain; 2 Institute of Agrochemistry and Food Technology (IATA), Spanish National Research Council (CSIC), Agustí Escardino 7, 46980 Paterna, Valencia Spain

## Abstract

Ochratoxin A is a potent toxic fungal metabolite whose undesirable presence in food commodities constitutes a problem of public health, so it is strictly regulated and controlled. For the first time, two derivatives of ochratoxin A (OTA*b* and OTA*d*) functionalized through positions other than the native carboxyl group of the mycotoxin, have been synthesized in order to better mimic, during the immunization process, the steric and conformational properties of the target analyte. Additionally, two conventional haptens making use of that native carboxyl group for protein coupling (OTA*e* and OTA*f*) were also prepared as controls for the purpose of comparison. The immunological performance in rabbits of protein conjugates based on OTA*b* and OTA*d* overcome that of conjugates employing OTA*e* and OTA*f* as haptens. After immunization of mice with OTA*b* and OTA*d* conjugates, a collection of high-affinity monoclonal antibodies to ochratoxin A was generated. In particular, one of those antibodies, the so-called OTA*b*#311, is very likely the best antibody produced so far in terms of selectivity and affinity to ochratoxin A.

## Introduction

**Figure 1:**
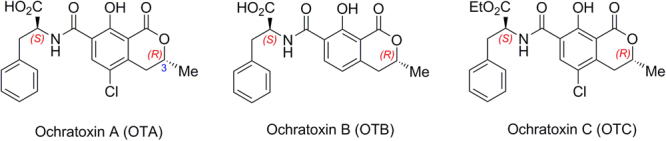
Chemical structures of the three ochratoxins.

Mycotoxins are secondary metabolites produced by a variety of fungi species that can contaminate agricultural and food commodities during the production, processing, storage, or distribution processes^1–3^. Among them, ochratoxins constitute a prominent three-member family of mycotoxins, all of them secreted by different species of the *Aspergillus* and *Penicillium* genera. Ochratoxin A (OTA) is undoubtedly the most significant toxin of this group, being ochratoxin B (OTB) and ochratoxin C (OTC) of lower relevance (Fig. 1). The only structural difference among them is the absence of the dihydroisocoumarin chlorine atom in OTB and the presence of a ethyl ester group in the L-phenylalanine moiety in the case of OTC. OTA has been shown to be a strong carcinogen for rodents and, since long ago, it is classified into group 2B – possibly carcinogenic to humans – by the International Agency for Research on Cancer (IARC)^4^. It is particularly nephrotoxic, and teratogenic, and immunotoxic effects have also been attributed to this substance^5,6^. This mycotoxin can be present in different foodstuffs, particularly in cereals but also in beverages such as coffee, beer, and wine^7–9^. Moreover, since OTA is very chemically stable, it remains unaltered during food processing^10^. For all those reasons, the European Food Safety Authority established a tolerable weekly intake value of 120 ng/kg of body weight for this mycotoxin^11^.

In accordance with its toxicological relevance, a great number of analytical methods have been developed to determine OTA^12^. High-performance liquid chromatography with fluorescence detection after immunoaffinity column clean-up is the most widely used analytical method for OTA, and the procedure recommended by the European Committee for Standardization, even though mass spectrometry detection is also widely employed^13–16^. With regard to receptor-based assays, although aptamers, binding peptides, and molecularly imprinted polymers have been developed in the last years^17–19^, methods based on antibodies as the recognition element, like the enzyme-linked immunosorbent assay (ELISA)^20–23^, immunochromatographic strips^24–26^, and immunosensors^27^, currently enjoy a high degree of implementation in quality control laboratories worldwide^28^. The key element of these methods is the antibody, because it is responsible for the sensitive and selective detection of OTA. Like any other small chemical compound, OTA is not immunogenic by itself, *i.e*., it does not trigger an immune response when administered to experimental animals. The strategy to generate antibodies to this kind of molecules, so-called haptens, consists on the preparation of covalent conjugates of the target compound with an immunogenic macromolecule, such as a protein. Consequently, a reactive or activatable chemical group needs to be present in the antigen. This is the case of OTA, which contains a carboxyl group that can be readily activated for protein conjugation. In fact, as far as we know, in all of the published research studies reporting the generation of monoclonal or polyclonal antibodies to OTA, the mycotoxin itself was covalently linked through its own carboxyl group to a carrier in order to prepare hapten‒protein conjugates for immunization^29–32^. However, this strategy, very likely employed because of its simplicity, may not be the most convenient from an immunological point of view. Theoretical calculations have revealed the great importance of the carboxylic acid moiety in the conformational arrangement of OTA^33,34^. Moreover, this chemical group can be envisioned as a main antigenic determinant for shaping the antigen binding site during the immune response. Considering these premises, the aim of the present study was to investigate whether functionalized OTA derivatives that keep free the native carboxyl group of OTA possess enhanced properties, in terms of immunogenic activity, for the generation of high-affinity antibodies. With this purpose, haptens with alternative linker tethering sites were designed and the immune response, regarding affinity and specificity, was assessed in rabbits. The obtained results were used to select the most appropriate conjugates for the generation of mouse monoclonal antibodies specific to OTA.

## Results and Discussion

### Hapten design and synthesis

**Figure 2:**
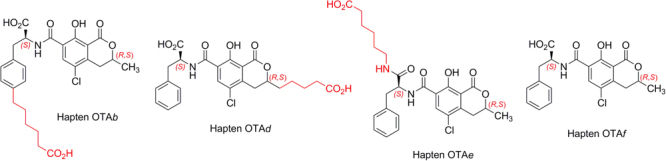
Chemical structures of the synthetic haptens.

As mentioned above, the generation of antibodies, both monoclonal and polyclonal, against OTA has been based so far on the use of immunogens prepared from the direct conjugation of OTA itself to a carrier protein. In all previous studies, this conjugation has been carried out through the formation of an amide linkage between the OTA native carboxyl group and protein amino groups. However, the conjugation of OTA through the carboxyl group may condition (constrain) the ability of the immunogen to adequately mimic the target molecule during the immune response. The derivatization of the carboxyl group introduces steric factors and limits the capacity of the molecule for intramolecular hydrogen-bonding formation, thus substantially modifying the conformational properties of the OTA framework. Moreover, the modification of a highly immunogenic functional group, such as the (carboxymethyl)carbamoyl group (HO_2_C─CH─NHCO─), may have important effects in the antibody-antigen interaction itself. In this work, we designed two haptens with alternative linker tethering sites that allow the conjugation of the complete OTA framework by distal positions, keeping free the native carboxyl group of OTA (Fig. 2). The first hapten (OTA*b*), incorporated a carboxylated hydrocarbon chain at the C-4 position of the benzene ring, while in the second one (OTA*d*) the carboxylated spacer arm was located at the C-3 position of the dihydroisocoumarin ring, which represents a formal homologation of the methyl group of OTA at this position. In both cases, the hapten handle did not imply a substantial modification in size, electronic and shape (conformation) properties of the target OTA core. Additionally, two conventional haptens making use of the native carboxyl group for protein coupling (OTA*e* and OTA*f*) were also prepared for comparison (Fig. 2), the latter with a linker equivalent in length to the one used in the novel haptens.

**Figure 3:**
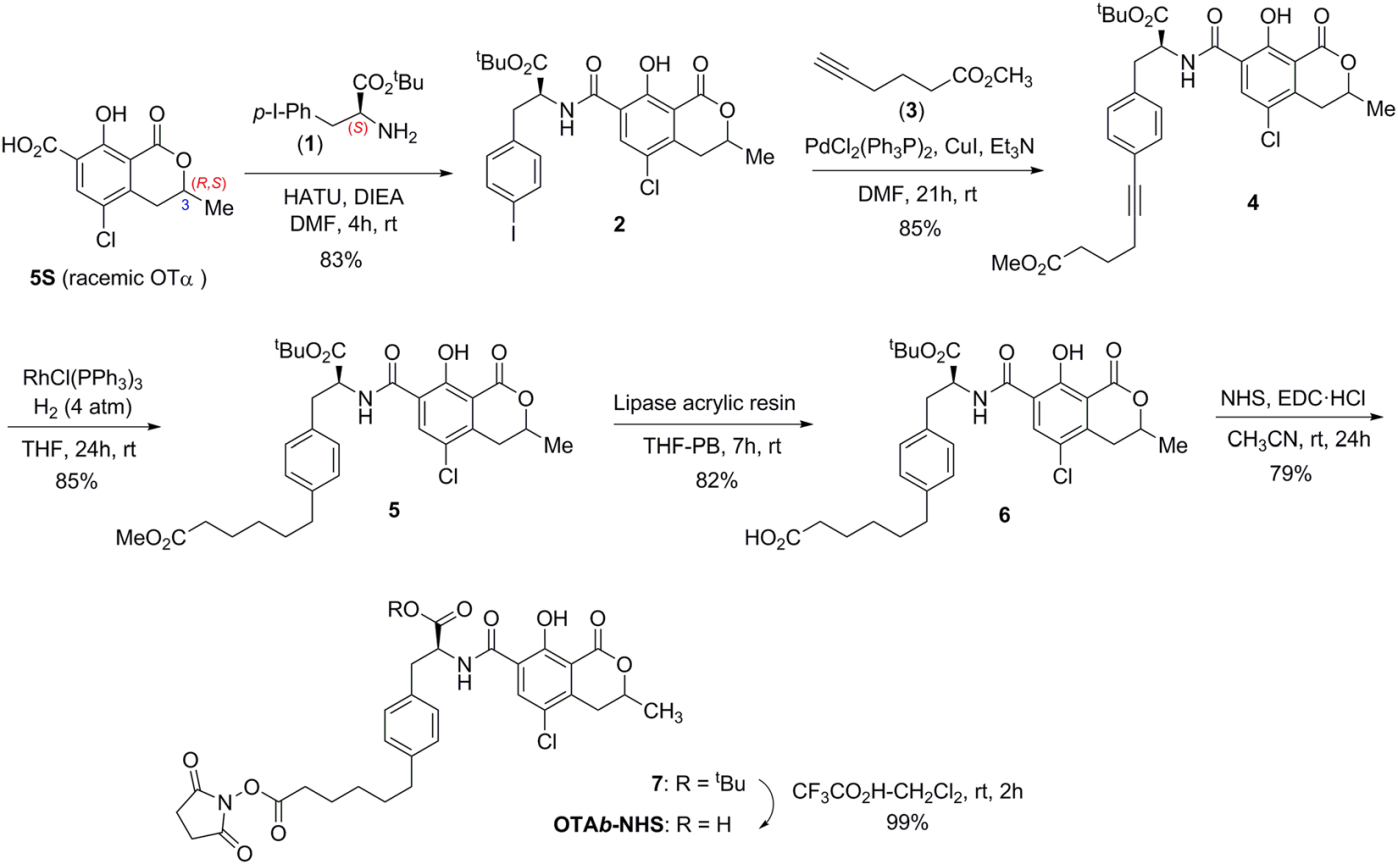
Synthetic route for the preparation of the NHS-ester of hapten OTA*b*.

As in the synthesis of OTA itself ^35–37^, preparation of all of the haptens followed a similar strategy for the construction of the common OTA core based on the initial preparation of conveniently functionalized L-phenylalanine and dihydroisocoumarin moieties, which are joined together through an amide bond. Thus, the synthesis of hapten OTA*b* started with the condensation of (*S*)-*tert*-butyl 2-amino-3-(4-iodophenyl)propanoate (**1**) with the dihydroisocumarin-7-carboxylic acid derivative 5S, also known as OTα, a major metabolite of OTA (Fig. 3)^38^. Although the stereocenter at the dihydroisocoumarin structure of OTA, *i.e*. C-3, has the (*R*)-configuration, we carried out the preparation of the required dihydroisocoumarin synthon **5**S in the synthetically most easily accessible racemic form (racemic OTα). Obviously, the use of this racemic synthon leaded to hapten OTA*b* as a mixture of diastereoisomers, epimers at the C-3 position of the dihydroisocoumarin ring.

Various synthetic approaches have been described in the literature for the preparation of racemic OTα^39^. In the present study, we have developed a new and more efficient procedure from *p*-chlorocresol whose details are found in the Supplementary Information file (Fig. S1). The condensation reaction between racemic OTα (**5**S) and protected 4-iodo-L-phenylalanine **1** was performed in presence of the uronium reagent HATU as coupling agent, to give a mixture of diastereoisomeric aryl iodides **2**. Once the core of the hapten common to OTA was completed, the carboxylated linker was introduced at the C-4 position of the phenyl ring subunit by means of a palladium catalyzed Sonogashira coupling reaction with methyl hex-5-ynoate (**3**) to give alkyne **4**. Hydrogenation of the acetylenic triple bond under homogeneous conditions using Wilkinson’s catalyst and chemoselective hydrolysis of the methyl ester group, using lipase from *Candida antarctica* immobilized on acrylic resin, completed the introduction of the carboxylic acid-terminated linker chain. Finally, the synthesis of hapten OTA*b*, in the form of active ester ready for its conjugation to the carrier proteins, was completed in two additional steps. First, activation of the carboxylic acid group of **6** via formation of the corresponding *N-*hydroxysuccinimidyl ester (**7**) by carbodiimide-mediated esterification with *N*-hydroxysuccinimide (NHS), and then chemoselective hydrolysis of the *tert*-butyl ester acid protecting group using trifluoroacetic acid. The six-step synthesis of OTA*b*-NHS ester from OTα was completed with an overall yield of about 38%.

**Figure 4:**
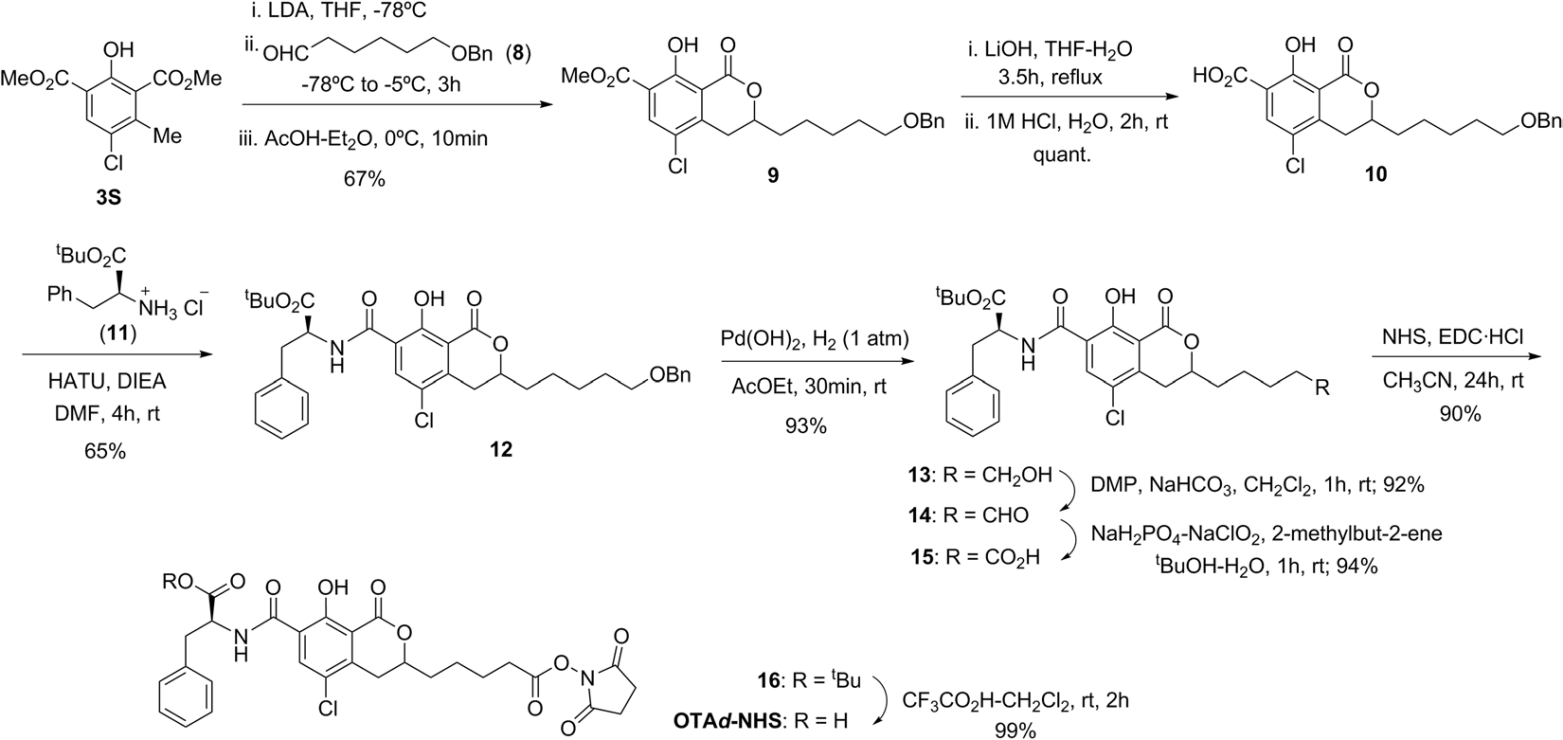
Synthetic route for the preparation of the NHS-ester of hapten OTA*d*.

As shown in Fig. 4, the synthesis of hapten OTA*d* also began with the construction of the dihydroisocoumarin moiety, following a similar strategy to that previously employed to synthesize racemic OTα. In this case, metalation of the methyl group of compound **3**S with excess of lithium diisopropylamide (LDA), followed by reaction of the benzylic anion generated with aldehyde **8** and acidic work-up, led to dihydroisocoumarin derivative **9**, which already incorporated the C5 hydrocarbon chain that constituted the spacer arm of the target hapten at the C-3 position. Basic hydrolysis of the methyl ester group and condensation reaction between the resulting carboxylic acid **10** and protected L-phenylalanine **11** completed the construction of the total carbon skeleton of this hapten. The rest of transformations that concluded its synthesis from intermediate **12** implied simple functional group interconversions. First, conversion of the linker chain terminal carbon atom into a carboxylic acid group via chemoselective hydrogenolitic cleavage of the benzyl protecting group to give the free alcohol **13**, followed by Dess-Martin periodinane (DMP) oxidation to aldehyde **14** and subsequent oxidation to the carboxylic acid using sodium chlorite. Finally, activation of the terminal carboxylic acid group of **15**, by conversion to the corresponding *N-*hydroxysuccinimidyl ester, and acid-catalyzed hydrolysis of the *tert*-butyl ester group, completed the synthesis of hapten OTA*d*, also in the form of active ester (*i.e*. OTA*d*-NHS) as a 1:1 mixture of epimers at the C-3 position of the dihydroisocoumarin moiety. The synthesis of OTA*d*-NHS required only eight steps from phenyl derivative **3**S and proceeded in 31% overall yield.

**Figure 5:**
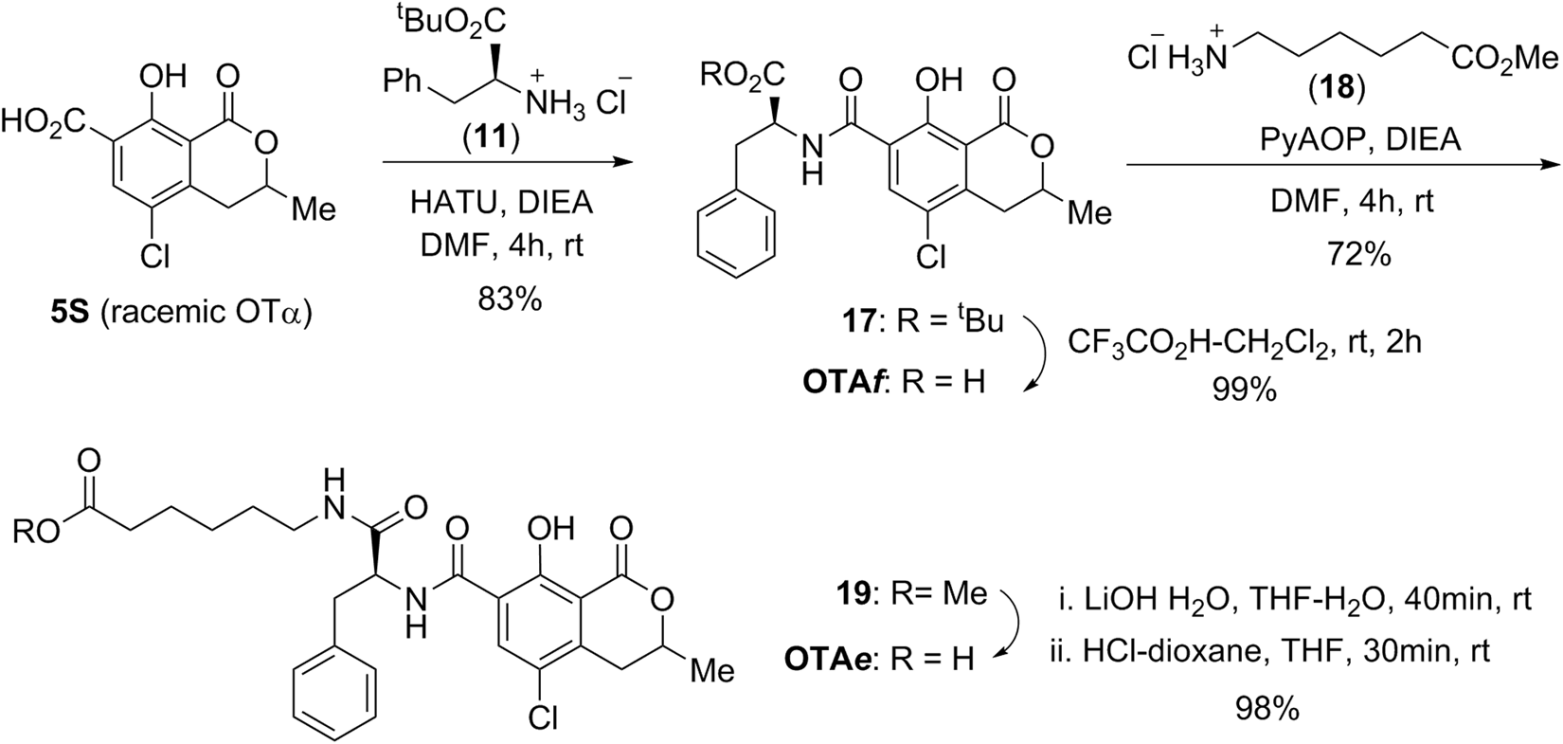
Synthetic route for the preparation of the haptens OTA*e* and OTA*f*.

The synthesis of hapten OTA*e* was very straightforward. It began with the preparation of *OTAf* by condensation of racemic OTα (**5**S) with protected L-phenylalanine (**11**) and subsequent acid hydrolysis of the *tert*-butyl ester group (Fig. 5). Next, the amino carboxylate linker was attached to the carboxylic acid group of *OTAf* by PyAOP-mediated coupling with amino-ester **18**, which was followed by chemoselective basic hydrolysis of the methyl ester group of **19** and brief acid treatment, to reverse the partial opening of the lactone ring caused by the basic hydrolytic treatment. The synthesis of hapten OTA*e* from OTα comprised only a 4-step sequence that proceeded in an overall yield of approximately 58%.

### Hapten activation and bioconjugate preparation

As mentioned above, haptens OTA*b* and OTA*d* were obtained with the functional group already activated in the form of *N*-hydroxysuccinimidyl ester and, therefore, ready for their conjugation to the proteins. Haptens OTA*e* and OTA*f*, initially synthetized in the free acid form, were transformed into the corresponding *N*-hydroxysuccinimidyl esters (OTA*e*-NHS and OTA*f*-NHS esters) using the carbodiimide-mediated esterification with NHS (see the Supplementary Information file). Every synthetized hapten was conjugated to bovine serum albumin (BSA), ovalbumin (OVA), and horseradish peroxidase (HRP). Using active esters – essentially pure and fully characterized – for the preparation of protein bioconjugates prevented the occurrence of side reactions during conjugation and allowed application of the same coupling procedure for immunizing and assay conjugates. In addition, the final hapten-to-protein molar ratio (MR) could be finely tuned in order to reach the desired MR values, usually over 10 for the immunizing conjugates, below 5 for coating conjugates, and around 1 for enzyme tracers. In the present study, the obtained MR values for BSA conjugates were between 10 and 15, between 2 and 4 for OVA conjugates and about 1 for HRP conjugates (Table 1 and Figs S2 to S4). The abnormally high MR value of the OVA‒OTA*b* conjugate suggests an incorrect estimation probably due to a deficient desorption from the plate during MALDI-TOF analysis, a circumstance previously observed by our group with some other OVA–hapten conjugates.

**Table 1:** Hapten-to-protein molar ratios of bioconjugates.

	MW	BSA		OVA		HRP	
		ΔMW^a^	MR^b^	ΔMW	MR	ΔMW	MR
OTA*b*	501	7560	15.1	2517	10.0	525	1.1
OTA*d*	472	5554	11.8	731	3.0	272	0.6
OTA*e*	500	7214	14.4	739	3.1	454	0.9
OTA*f*	385	4139	10.8	205	1.1	326	0.8

^a^Increment of the protein molecular weight. ^b^Molar ratio.

### Evaluation of hapten immunogenicity

In order to evaluate the immunogenicity of OTA haptens, antisera were generated and analyzed because they constitute a direct depiction of the response of the immune system to the immunogen. Antisera from immunized rabbits were assayed by competitive ELISA using the capture antibody-coated direct format in order to avoid biased results due to avidity effects or antibody conformational changes that may occur with other competitive ELISA formats. Immunoassays were carried out using the homologous enzyme tracer, *i*.*e*., with HRP coupled to the same hapten as the immunizing conjugate. Unlike the results reported by different research groups^40–42^, no signal was obtained when antisera from hapten OTA*f* were combined with the homologous enzyme tracer (HRP‒OTA*f*). Poor or no binding between antibodies and enzyme tracers based on haptens without or with short spacer arm has been previously observed by our group and others for a variety of analytes^43–45^. Insufficient signal could be a consequence of either no binding because of steric hindrances or a result of enzyme inactivation due to the close proximity of the antibody to the label. However, we observed that the peroxidase activity was maintained in a mixture in buffer between the antibody and the homologous enzyme tracer, so inaccessibility of the hapten seems to be the most feasible hypothesis. In order to test this idea, the OTA*f*-type antiserum was assayed with the HRP–OTA*e* tracer, which holds the linker at the carboxyl group of OTA, thus resembling the spatial orientation, conformation, and electronic density of the homologous OTA*f* enzyme tracer. Under these conditions, strong signals were obtained, so the tracer HRP–OTA*e* was further used for the characterization of OTA*f*-derived antisera.

**Figure 6:**
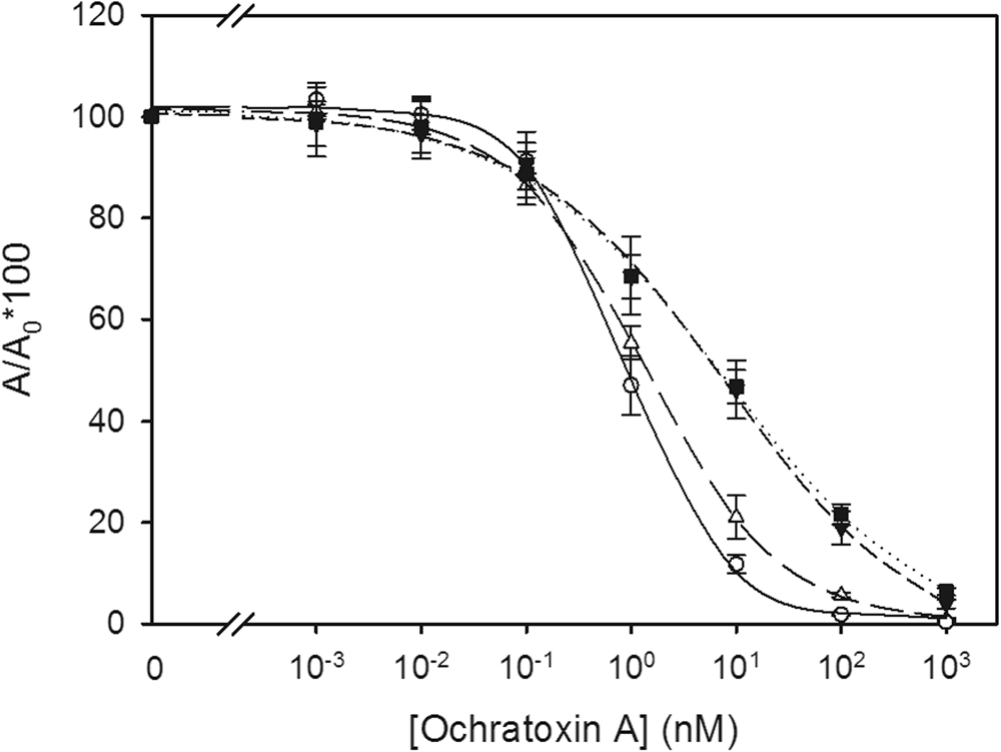
OTA inhibition curves obtained with the four types of rabbit antisera: OTA*b* (open triangles), OTA*d* (open circles), OTA*e* (black triangles), and OTA*f* (black squares). IC_50_ values were 1.32, 0.86, 8.20, and 7.91 nM, respectively. A_max_ values were higher than 1.5 and the background asymptotes were close to zero.

Characterization of the rabbit antisera by competitive ELISA revealed that antibodies raised from haptens OTA*b* and OTA*d* ‒ with the native OTA carboxyl group in free form ‒ exhibited higher affinity to OTA than the antibodies generated from haptens OTA*e* and OTA*f*, in which the carboxyl group was blocked (Fig. 6). In particular, the IC_50_ values for OTA with OTA*b*-type and OTA*d*-type antisera were around 8-fold lower than those with OTA*e*-type and OTA*f*-type antisera (1 nM *vs* 8 nM). Moreover, steeper slopes were observed for the former than for the latter. The best inhibition curve (lowest IC_50_ value and slope closer to 1.0) was achieved using the antiserum that was obtained with the hapten OTA*d*, the derivative holding the spacer arm at a distal position from the carboxylic acid moiety. In addition, the inhibition curve of OTA*e*-type antiserum was equivalent to that of OTA*f*-type antiserum, meaning that the presence of a linker was not so important for this coupling position. These results support our initial hypothesis asserting that the carboxyl group of OTA is a relevant antigenic determinant moiety, as demonstrated by the enhanced immunogenic performance of conjugates bearing haptens that keep free this characteristic substituent over the classical approach that blocks this group.

The tethering site of the spacer arm also influenced largely the specificity of the generated antibodies. On the one hand, none of the antisera was able to significantly recognize OTB, thus pointing out the strong influence of the OTA chlorine atom in the antibody‒antigen interaction (Table 2). However, on the other hand, whereas antisera raised from haptens OTA*b* and OTA*d* – with free carboxyl group – were not able to bind OTC, antibodies from haptens OTA*e* and OTA*f* – with the native OTA carboxyl group blocked – recognized this analogue toxin even much better than OTA (the IC_50_ values were 4 or even 20 times lower for the former toxin than for the latter). This finding was not surprising because, from a molecular point of view, OTA*e* and OTA*f* are more appropriate derivatives for the generation of anti-OTC antibodies than for the production of anti-OTA binders. Therefore, hapten functionalization at positions other than the native carboxyl moiety present in OTA, like in haptens OTA*b* and OTA*d*, not only elicited a superior affinity immune response to the mycotoxin, but also triggered a more specific response.

**Table 2:** Cross-reactivity (%) of polyclonal antibodies to other ochratoxins.

	OTB	OTC
OTA*b*#1	2.4	0.1
OTA*b*#2	3.1	0.2
OTA*d*#1	1.4	0.1
OTA*d*#2	2.4	0.1
OTA*e*#1	0.7	465.0
OTA*e*#2	5.7	2219.6
OTA*f*#1	0.5	449.9
OTA*f*#2	0.9	432.8

### Generation of monoclonal antibodies

To further assess whether monoclonal antibodies able to tightly bind OTA can be raised from haptens that keep unmodified the characteristic carboxylic acid moiety, mice were immunized with the BSA conjugates of haptens OTA*b* and OTA*d*, and a collection of hybridomas was selected and cloned. Performance of the purified monoclonal antibodies was assessed by checkerboard titration in the antibody-coated direct competitive ELISA format using the homologous conjugates (Table 3). No significant differences were observed between both types of antibodies. Nearly all monoclonals showed IC_50_ values to the mycotoxin below 1 nM, and two of them displayed even higher affinity, with IC_50_ values below 0.1 nM. Once more, these results confirm the efficiency of these haptens to generate monoclonal antibodies with an outstanding ability to recognize OTA.

**Table 3:** Characterization of mouse monoclonal antibodies (n = 3).

mAb	[mAb]^a^	[HRP]^a^	A_max_	Slope	IC_50_^b^
OTA*b*#39	100	10	1.880	1.309	0.50
OTA*b*#41	100	10	0.837	0.860	1.14
OTA*b*#310	100	30	1.103	1.116	0.09
OTA*b*#311	100	30	1.054	1.324	0.07
OTA*d*#16	300	10	0.959	1.876	0.74
OTA*d*#21	300	10	0.982	1.892	0.75
OTA*d*#27	100	10	0.826	1.150	0.75
OTA*d*#111	300	10	1.071	2.173	0.88
OTA*d*#114	100	10	0.881	1.480	0.50
OTA*d*#115	100	100	1.532	1.348	0.14
OTA*d*#118	100	30	1.198	1.893	0.30

^a^Antibody and enzyme tracer concentration values are in ng/mL. ^b^Values are in nM units.

Concerning specificity, none of the OTA*b*-type monoclonal antibodies significantly recognized OTB or OTC, showing cross-reactivity values around or below 1% (Table 4). Interestingly, the specificity of OTA*d*-type monoclonal antibodies was varied. Regarding OTB, some antibodies showed low or no binding, whereas other antibodies displayed moderate to good recognition of this compound, indicating a dissimilar behavior with polyclonal antibodies. On the other hand, the cross-reactivity towards OTC was mostly quite low. Therefore, the capacity to bind OTB – without the chlorine atom – was determined by the position of the linker in the immunizing hapten. On the contrary, the position of the spacer was not relevant for the recognition of OTC, in which the carboxyl group is esterified and therefore not freely available for the interaction with the antibody binding site. Accordingly, it seems that the chlorine group was a relevant antigenic determinant but not as much as the carboxylic acid moiety.

**Table 4:** Cross-reactivity (%) of monoclonal antibodies to other ochratoxins.

	OTB	OTC
OTA*b*#39	−^a^	—
OTA*b*#41	1.0	1.1
OTA*b*#310	0.7	—
OTA*b*#311	1.1	0.3
OTA*d*#16	1.7	—
OTA*d*#21	2.2	—
OTA*d*#27	—	—
OTA*d*#111	87.9	0.6
OTA*d*#114	—	—
OTA*d*#115	58.7	1.3
OTA*d*#118	18.1	—

^a^Cross-reactivity was lower than 0.1%.

In conclusion, through efficient multi-step synthetic procedures, two novel OTA derivatives have been prepared with a spacer arm located at sites of the mycotoxin framework that had not been explored previously. The linker-tethering position has been shown to influence both the affinity and the specificity of the generated antibodies. Antisera obtained with conjugates of haptens that kept free the native carboxylic acid group of OTA displayed higher affinity than those obtained by the classical approach. Accordingly, carboxyl groups constitute highly relevant antigenic determinants, so synthetic efforts aimed at keeping them unmodified are certainly worthy for analytical targets bearing this sort of functional group. Following optimization of assay conditions, the monoclonal antibodies herein described, in particular OTA*b*#311, arise as very promising reagents for the development of immunoanalytical methods with outstanding sensitivity for the determination of OTA contamination in a variety of foodstuffs.

## Materials and Methods

### Hapten synthesis and activation

Details about chemicals, general experimental techniques, equipment, and full experimental details and thorough physical and spectroscopic data of the haptens and all of the intermediates of the synthesis are given in the Supplementary Information file. Haptens OTA*b* and OTA*d* were directly obtained as the corresponding *N*-hydroxysuccinimidyl active esters (OTA*b*-NHS and OTA*d*-NHS) according to the synthetic routes detailed in Figs 3 and 4. Haptens OTA*e* and OTA*f* (an equimolecular mixture of OTA and its epimer at the dihydroisocoumarin C-3 position) were prepared as shown in Fig. 5. They were transformed into the corresponding active ester (OTA*e*-NHS and OTA*f*-NHS) for coupling to the proteins by using the carbodiimide-mediated esterification with NHS. The basic characterization data of each of the four active esters are given below.

Compound OTA*b*-NHS was obtained as a resinous and brownish material (a 1:1 mixture of diastereoisomers). ^1^H NMR (CDCl_3_, 500 MHz) δ (ppm) 1.42 (m, 2 H), 1.57–1.68 (m, 2 H), 1.60 (two d, each 1.5 H, *J* = 6.4 Hz), 1.75 (m, 2 H), 2.59 (m, 4 H), 2.78–2.91 (m, 5 H), 3.16–3.34 (m, 3 H), 4.77 (m, 1 H), 5.00–5.04 (m, 1 H), 7.06–7.18 (m, 4 H), 8.42 (m, 1 H), 8.51 and 8.58 (two m, each 0.5 H); HRMS (TOF ESI+) calcd for C_30_H_32_ClN_2_O_10_ [M + H]^+^ 615.1740, found 615.1732.

Compound OTA*d*-NHS was obtained as a resinous, brownish-colored residue (a 1:1 mixture of diastereoisomers)^1^.H NMR (CDCl_3_, 300 MHz) δ (ppm) 1.54–1.93 (m, 6 H), 2.68 (t, *J* = 6.69 Hz, 2 H), 2.85 (br s, 4 H), 2.92 (m, 1 H), 3.17–3.43 (m, 3 H), 4.61 (m, 1 H), 5.03 (m, 1 H), 7.18–7.33 (m, 5 H), 8.39 (s, 1 H), 8.56 (br s, 1 H), 12.73 (br s, 1 H); HRMS (TOF ESI + ) calcd for C_28_H_28_ClN_2_O_10_ [M + H]^+^ 587.1427, found 587.1425.

Compound OTA*e*-NHS was obtained as a yellowish oil (a 1:1 mixture of diastereoisomers)^1^.H NMR (DMSO-*d*_6_, 500 MHz) δ (ppm) 1.29–1.37 (m, 2 H), 1.39–1.47 (m, 2 H), 1.60 (d, *J* = 6.4 Hz, 3 H), 1.64–1.78 (m, 2 H), 2.56 (t, *J* = 6.8 Hz, 2 H), 2.76–2.93 (m, 5 H), 3.06–3.37 (m, 5 H), 4.68–4.91 (m, 2 H), 6.04 (m, 1 H), 7.16–7.35 (m, 5 H), 8.38 (s, 1 H), 8.60 (d, *J* = 7.0 Hz, 1 H), 12.79 (s, 1 H); HRMS (TOF ESI + ) calcd for C_30_H_33_N_3_ClO_9_ [M + H]^+^ 614.1900, found 614.1892.

Compound OTA*f*-NHS was obtained as a viscous oil (a 1:1 mixture of diastereoisomers)^1^.H NMR (CDCl_3_, 300 MHz) δ (ppm) 1.54–1.61 (d, *J* = 6.4 Hz, 3 H, Me-3′), 2.78–2.90 (m, 5 H, H-4′, COCH_2_CH_2_CO), 3.22–3.37 (m, 2 H, H_2_–3), 3.40–3.51 (m, 1 H, H-4′), 4.74 (m, 1 H, H-3′), 5.30–5.42 (m, 1 H, H-2), 7.21–7.39 (m, 5 H, Ph), 8.37–8.48 (m, 2 H, H-6′ and NH), 12.70 (s, 1 H, OH); HRMS (TOF ESI + ) calcd for C_20_H_17_ClNO_5_ [M‒C_4_H_4_NO_3_]^+^ 386.0790, found 386.0785.

### Conjugation to proteins

Coupling was performed in 50 mM carbonate‒bicarbonate buffer, pH 9.6, during 2 h at rt. A 50 mM solution of the purified NHS ester of hapten OTA*b*, OTA*d*, OTA*e*, or OTA*f* in *N*,*N*-dimethylformamide was dropwise added over a 15 mg/mL BSA or OVA solution in coupling buffer under moderate stirring. Immunizing conjugates were prepared by mixing 24 µmol of the corresponding activated hapten per micromole of BSA. Coating conjugates of the same haptens were prepared by adding 8 µmol of the corresponding active ester per micromole of OVA. For enzyme tracer preparation, 5 mM hapten‒NHS ester solutions in *N*,*N*-dimethylformamide were gently and slowly mixed with a 2.5 mg/mL HRP solution in coupling buffer. In this case, 8 µmol of hapten active ester per micromole of peroxidase was used. All conjugates were purified by size exclusion chromatography using a 15 mL Sephadex G-25 HiTrap Desalting Column from GE Healthcare (Uppsala, Sweden) and 100 mM phosphate buffer, pH 7.4, as eluent. Coupling extents were calculated by Matrix-Assisted Laser Desorption Ionization Time-of-Flight Mass Spectrometry (MALDI-TOF/MS) with a 5800 MALDI-TOF-TOF (ABSciex, Framingham, MA) apparatus, and running BSA, OVA, and HRP as references in the same plate. Further details about sample preparation and MALDI analysis are described in the Supplementary Information file.

### Antibody generation

Experimental design was approved by the Bioethics Committee of the University of Valencia. Animal manipulation was performed in compliance with the European Directive 2010/63/EU and the Spanish laws and guidelines (RD1201/2005 and 32/2007) concerning the protection of animals used for scientific purposes. Four groups of two female New Zealand white rabbits were immunized by subcutaneous injection either with BSA‒OTA*b*, BSA‒OTA*d*, BSA‒OTA*e*, or BSA‒OTA*f* conjugate and, ten days after the fourth injection, rabbits were exsanguinated. The eight obtained antibodies were precipitated twice with a 3.90 M ammonium sulfate solution and the precipitates were stored at 4 °C. Antibodies were characterized by checkerboard competitive ELISA using the homologous enzyme tracer. Additional information is provided in the Supplementary Information file.

For monoclonal antibody generation, two sets of four mice were immunized by intraperitoneal injection of BSA‒OTA*b* or BSA‒OTA*d* conjugate, and hybridomas were prepared by fusion of mouse myeloma cells with B lymphocytes from two equally-immunized mice as published elsewhere^46^. Antibody-producing cells were screened by a double sequential procedure as described in the Supplementary Information file. Monoclonal antibodies were purified from late stationary phase hybridoma cell cultures by affinity chromatography and stored at 4 °C as ammonium sulfate precipitates. Antibodies were characterized by checkerboard competitive ELISA using homologous enzyme tracers. See the Supplementary Information file for additional information about this section.

### Competitive ELISAs

Microwells were coated by overnight incubation at 4 °C with 100 µL per well of polyclonal goat anti-rabbit immunoglobulins or goat anti-mouse immunoglobulins (2.2 µg/mL) in 50 mM carbonate‒bicarbonate buffer, pH 9.6. After washing the plates four times with 150 mM NaCl solution containing 0.05% (v/v) Tween 20, 100 µL per well of antiserum or monoclonal antibody solution was applied and plates were incubated 1 h at room temperature. Then, plates were washed as previously and 50 µL per well of OTA solution in PBS (10 mM phosphate buffer, pH 7.4, with 140 mM NaCl) plus 50 µL per well of enzyme tracer solution in PBST (PBS containing 0.05% (v/v) Tween 20) were mixed and incubated 1 h at room temperature. Finally, plates were washed again and the retained peroxidase activity was revealed during 10 min at room temperature by adding 100 µL per well of *o*-phenylendiamine solution (2 mg/mL) in 25 mM citrate and 62 mM phosphate buffer, pH 5.4, containing 0.012% (v/v) H_2_O_2_. The reaction was stopped with 100 µL per well of 1 M H_2_SO_4_.

A concentrated mycotoxin solution was prepared from a stock solution in *N*,*N*-dimethylformamide by 1000-fold dilution in PBS. The standard curve consisted of seven mycotoxin solutions at different concentrations, plus a blank, which were prepared in borosilicate glass vials by serial dilution in PBS, starting from the freshly prepared concentrated solution in buffer. After the assay was concluded, ELISA absorbances at 492 nm were immediately read using a reference wavelength at 650 nm. Experimental values were fitted with the SigmaPlot software from SPSS Inc. (Chicago, IL, USA) to a four-parameter logistic equation. The OTA concentration at the inflexion point of the sigmoidal curve was taken as the assay sensitivity, which typically corresponds to a 50% reduction (IC_50_) of the maximum signal (A_max_). Cross-reactivity was calculated as the percentage value of the quotient between the IC_50_ for OTA and the IC_50_ for the other mycotoxin.

### Material Availability

Upon signing of a material transfer agreement, limited amounts of the monoclonal antibodies and bioconjugates herein described are available for evaluation upon request to the corresponding authors.

## Electronic supplementary material

Supplementary Information
