# Supplementary material for: Novel haptens and monoclonal antibodies with subnanomolar affinity for a classical analytical target, ochratoxin A
Source: Sci Rep. 2018 Jun 27;8:9761. doi: 10.1038/s41598-018-28138-x (PMC6021394; doi:10.1038/s41598-018-28138-x)
Supplement: Supplementary file 1 — Supplementary Information [file 41598_2018_28138_MOESM1_ESM.pdf]

SUPPLEMENTARY INFORMATION

Novel haptens and monoclonal antibodies with subnanomolar affinity for a classical analytical target, ochratoxin A

Daniel López-Puertollano, Josep V. Mercader, Consuelo Agulló, Antonio Abad-Somovilla, Antonio Abad-Fuentes

Contents | **Page**

1. General experimental procedures and techniques	S1
2. Synthesis of racemic OTα	S2
3. Synthesis of the *N*-hydroxysuccinimidyl ester of hapten OTA*b*	S6
4. Synthesis of the *N*-hydroxysuccinimidyl ester of hapten OTA*d*	S11
5. Synthesis of the *N*-hydroxysuccinimidyl ester of hapten OTA*e* and OTA*f*	S17
6. Preparation of bioconjugates	S21
7. MALDI mass spectrometry analysis of bioconjugates	S22
8. Antibody generation	S24

1. General experimental procedures and techniques

1.1. Reagents, equipment, and general techniques used in the preparation of haptens and bioconjugates

Organic solvents were dried and distilled prior use using standard techniques.[1] Et_2O and THF were distilled over Na and benzophenone under N_2 atmosphere just before being used. CH_2Cl_2 and CH_3CN were distilled from CaH_2 in the same way. MeOH was dried and stored on activated molecular sieves (3Å). DMF was distilled from CaH_2 at 7 mmHg and stored at –20 °C on 4Å molecular sieve. The remaining solvents and commercial reagents were used without prior purification. The operations with air and/or moisture-sensitive reagents were carried out under an inert atmosphere of dry N_2 or Ar, using syringes and/or cannulas, oven-dried (130 °C) glass material and freshly distilled and dried solvents. Reactions were monitored by thin-layer chromatography (TLC) on precoated silica plates (0.25 mm layer thickness, Silica Gel 60 F_{254}) using UV light as the visualizing agent and ethanolic phosphomolybdic acid or aqueous ceric ammonium molybdate solutions and heat as developing agents. The synthesized compounds were purified by flash column chromatography using silica gel 60 (particle size 0.043–0.063 mm). Melting points were determined on a Büchi M-560 apparatus and are uncorrected. Proton and carbon nuclear magnetic resonance (^{1}H and ^{13}C NMR) spectra were recorded at room temperature (rt) on a Bruker Avance DPX300 spectrometer operating at 300.1 and 75.5 MHz, respectively, or on a Bruker Avance DRX500 spectrometer operating at 500.1 and 125.8 MHz, respectively. Chemical shifts (δ) are expressed in ppm recorded using the residual solvent as the internal reference in all cases [7.27/77.00 ppm and 2.50/39.51 for the ^{1}H/^{13}C spectra in $CDCl_3$ and DMSO, respectively (ACD/NMR Processor Academic Edition spectra processing program, version 12.0)]. Carbon substitution degrees were established by DEPT (Distortionless Enhancement by Polarisation Transfer) pulse sequences. A combination of COSY (COrrelation SpectroscopY) and HSQC (Heteronuclear Simple Quantum Coherence) experiments was used in most cases for the assignment of ^{1}H and ^{13}C chemical shifts. High resolution mass spectra (HRMS) were obtained by electrospray ionization (ESI) mode in a premier Q-TOF mass spectrometer equipped with an electrospray source (Waters, Manchester, UK). The obtained data are expressed as mass/charge ratio (*m/z*). Analysis of hapten bioconjugates was performed by mass spectrometry in a 5800 MALDI TOF-TOF (ABSciex) in positive linear mode (1500 shots for each position) in a mass range of 10000-120000 *m/z*.

[1] Perrin DD, Armarego WLF, in "Purification of Laboratory Chemicals", 4th ed.; Butterworth Heinemann Press: Oxford, 1996.

1.2. Reagents and equipment used in antibody generation and immunoassays

Ochratoxin A was purchased from Sigma/Aldrich (Madrid, Spain) and ochratoxins B and C from Toronto Research Chemicals (Ontario, Canada). BSA fraction V was from Roche Applied Science (Mannheim, Germany). OVA, HRP, adult bovine serum, Freund's adjuvants, and o-phenylenediamine were provided by Sigma/Aldrich (Madrid, Spain). Goat anti-rabbit immunoglobulins and goat anti-mouse immunoglobulins were obtained from Rockland Inc. (Limerick, PA, USA) and Jackson ImmunoResearch Laboratories Inc. (West Grove, PA, USA), respectively. As secondary antibody, polyclonal rabbit anti-mouse immunoglobulin antibody peroxidase conjugate from Dako (Glostrup, Denmark) was used. Costar flat-bottom high-binding 96-well polystyrene ELISA plates from Corning (Corning, NY, USA) were used. ELISA absorbances were read with a PowerWave HT from BioTek Instruments (Winooski, VT, USA). Microplate wells were washed with an ELx405 microplate washer also from BioTek Instruments.

2. Synthesis of racemic OTα (5S)

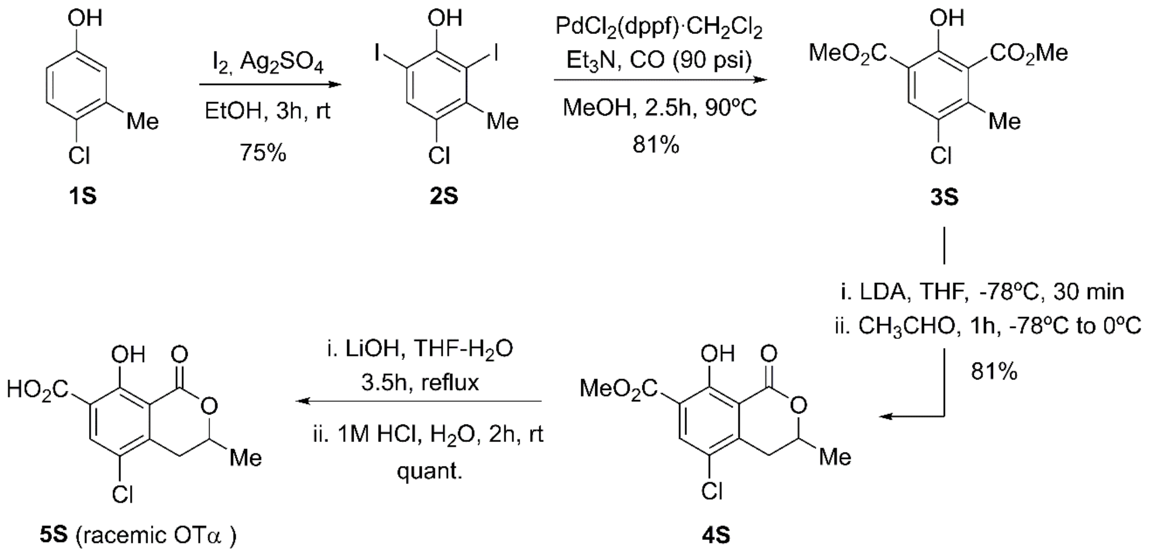

Figure S1. New synthetic route for the preparation of OTα

Racemic OTα (**5S**) shown in Figure S1 was prepared through a new synthetic route starting from 4-chloro-3-methylphenol (*p*-chlorocresol, **1S**), a very cheap industrial product widely used as an antiseptic and preservative. The synthesis began with the iodation of both positions *ortho* to the phenolic OH group to give the aryl diode **2S**, which upon treatment with CO/MeOH under palladium-catalyzed carbonylation reaction conditions afforded diester **3S**. The construction of the

dihydroisocoumarin ring was accomplished based on the Kraus's method,[2] which involves the deprotonation of the methyl group with lithium diisopropylamide (LDA) and subsequent capture of the generated benzylic anion with excess acetaldehyde. The synthesis of OTα was readily completed by basic hydrolysis of the methoxycarbonyl group to the corresponding carboxylic acid group.

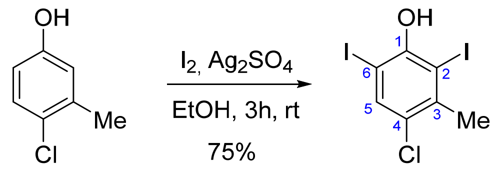

1S **2S**

2.1. **Preparation of 4-chloro-2,6-diiodo-3-methylphenol** (**2S**). Absolute EtOH (31 mL) was added to a mixture of 4-chloro-3-methylphenol (**1S**) (1.78 g, 12.5 mmol), iodine (6.93 g, 27.3 mmol, 2.2 equiv) and Ag_2SO_4 (7.76 g 24.9 mmol, 2 equiv) and the mixture was stirring for 3 h at rt. After the reaction was completed, the mixture was filtered to separate the salts, using $CHCl_3$ to wash. The filtrate was washed with a 10% aqueous solution of $Na_2S_2O_3$ and brine. After drying over anhydrous $MgSO_4$ and evaporation of the solvent in vacuo, the residue was purified by chromatography, using hexane as eluent, to obtain compound **2S** (3.72 g, 75%) as a white solid. Mp 91.0–92.0 °C (crystallized from cold hexane). ^{1}H NMR ($CDCl_3$, 300 MHz) δ (ppm) 2.59 (s, 3H, Me-3), 5.83 (s, 1H, OH), 7.72 (s, 1H, H-5); ^{13}C NMR ($CDCl_3$, 75 MHz) δ (ppm) 26.8 (Me-3), 77.6 (C-6), 90.5 (C-2), 125.2 (C-4), 137.9 (C-5), 140.3 (C-3), 152.6 (C-1); HRMS (TOF ESI–) calcd for $C_7H_4ClI_2O$ $[M-H]^-$ 392.8046, found 392.8042.

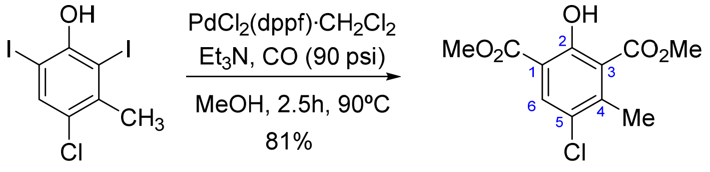

2S **3S**

2.2. **Preparation of dimethyl 5-chloro-2-hydroxy-4-methylbenzene-1,3-dicarboxylate** (**3S**). A mixture of diiodo phenol **2S** (300 mg, 0.76 mmol) and $PdCl_2(dppf)\cdot CH_2Cl_2$ (64.0 mg, 0.08 mmol, 0.1 equiv) in anhydrous MeOH (9 mL) contained in a Büchi 'Tiny Clave' reactor equipped with a magnetic stirring bar, was exhaustively degassed by vacuum-filling argon cycles and cooled to 0 °C. Then, Et_3N (0.53 mL, 3.8 mmol, 5 equiv) was quickly added under a stream of argon and the mixture re-subjected to pump/purge cycles at 0 °C, first with argon and then with CO. The CO pressure was set to 90 psi and the reaction mixture was stirred at 90 °C for 2.5 h. After this time the reactor was cooled and vented, the reaction mixture was transferred to a round-bottom flask with the aid of CH_2Cl_2 and concentrated to dryness under reduced pressure. The obtained residue was suspended

---

[2] Kraus, G. A. A Facile Synthesis of Ochratoxin A. *J. Org. Chem*, *46*, 201–202 (1981)

in Et_2O and filtered, the filtrate was washed with a 1M aqueous solution of HCl and brine and dried over anhydrous $MgSO_4$. The residue left after evaporation of the solvent was purified by chromatography on silica gel, using hexane-EtOAc mixtures (95: 5 and 90:10) as eluent, to give compound **3S** (158.4 mg, 81%) as a white solid. Mp 66.1–67.1 °C (crystallized from cold hexane). ^1H NMR ($CDCl_3$, 300 MHz) δ (ppm) 2.35 (s, 3H, Me-4), 3.96 (s, 6H, 2xOMe), 7.87 (s, 1H, H-6), 10.96 (s, 1H, OH); ^{13}C NMR ($CDCl_3$, 75 MHz) δ (ppm) 18.0 (Me-4), 52.7 (2xOCH_3), 111.5 (C-1), 124.9 (C-3), 125.1 (C-5), 130.6 (C-6), 141.5 (C-4), 156.8 (C-2), 166.8 (CO_2-1), 169.1 (CO_2-3); HRMS (TOF ESI+) calcd for $C_{11}H_{12}ClO_5$ $[M+H]^{\cdot+}$ 259.0368, found 259.0356.

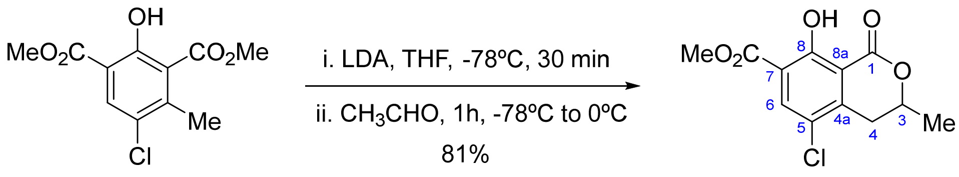

3S **4S**

2.3. **Preparation of methyl 5-chloro-8-hydroxy-3-methyl-1-oxoisochromane-7-carboxylate** (**4S**). A solution of dimethyl dicarboxylate **3S** (135.6 mg, 0.52 mmol) in anhydrous THF (260 µL) was added dropwise to a solution of LDA in THF [generated from diisopropylamine (193 µL, 1.36 mmol, 2.6 equiv), BuLi (822 µL of a 1.6 M solution in hexane, 1.31 mmol, 2.5 equiv) and anhydrous THF (1.5 mL)] at –78 °C under nitrogen. The orange mixture was stirred for 20-30 min at –78 °C and then dry acetaldehyde (400 µL, 315 mg, 7.15 mmol, 14 equiv) was added. The reaction mixture was stirred at the same temperature for 10 min and at 0 °C for 1 h. After this time, the resulting yellowish reaction mixture was quenched by the addition of a 1:2 solution of AcOH in Et_2O (1 mL), then diluted with EtO_2 and washed with water, a 5% aqueous solution of $NaHCO_3$ and brine, dried over anhydrous $MgSO_4$ and concentrated in vacuo. The obtained residue (192 mg) was purified by column chromatography, using mixtures of hexane-EtOAc (100:0 and 85:15) as eluent, to give compound **4S** (115.0 mg, 81%) as a white semi-solid. ^1H NMR ($CDCl_3$, 300 MHz) δ (ppm) 1.58 (d, J = 6.4 Hz, 3H, Me-3), 2.84 (dd, J = 17.4, 11.6 Hz, 1H, H-4), 3.27 (dd, J = 17.3, 3.2 Hz, 1H, H'-4), 3.95 (s, 3H, CO_2CH_3), 4.42-4.92 (m, 1H, H-3), 8.11 (s, 1H, H-6), 12.19 (s, 1H, OH); ^{13}C NMR ($CDCl_3$, 75 MHz) δ (ppm) 20.6 (Me-3), 32.6 (C-4), 52.6 (OCH_3), 75.1 (C-3), 111.2 (C-8a), 118.4 (C-7), 121.7 (C-5), 138.0 (C-6), 142.2 (C-4a), 161.1 (C-8), 165.0 (C-1), 167.9 (CO_2-7); HRMS (TOF ESI+) calcd for $C_{12}H_{12}ClO_5$ $[M+H]^{\cdot+}$ 271.0368, found 271.0371.

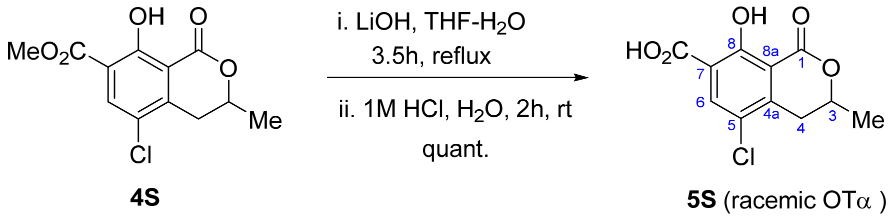

2.4. Preparation of 5-chloro-8-hydroxy-3-methyl-1-oxoisochromane-7-carboxylic acid (**5S**, racemic OTα). A solution of LiOH·H_2O (115.0 mg, 2.73 mmol, 10 equiv) in water (1.20 mL) was added to a suspension of dihydroisocoumarin **4S** (74.0 mg, 0.27 mmol) in anhydrous THF (910 µL). The mixture was refluxed for 3.5 h and then cooled to 0 °C and acidified with a 1M aqueous solution of HCl (4.91 mL, 4.91 mmol, 18 equiv). The reaction mixture was stirred at rt for 2 h, diluted with water and extracted with EtOAc. The combined organic phases were washed with brine, dried over anhydrous $MgSO_4$ and concentrated under reduced pressure to afford the acid **5S** (69.9 mg, nearly quantitative yield) as a light brown amorphous solid. ^{1}H NMR (DMSO-*d*$_6$, 300 MHz) δ (ppm) 1.44 (d, *J* = 6.2 Hz, 3H, Me-3), 2.88 (dd, *J* = 17.3, 11.6 Hz, 1H, H-4), 3.20 (dd, *J* = 17.3, 3.2 Hz, 1H, H'-4), 4.75 (m, 1H, H-3), 7.99 (s, 1H, H-6); ^{13}C NMR (DMSO-*d*$_6$, 75 MHz) δ (ppm) 20.1 (Me-3), 32.2 (C-4), 74.4 (C-3), 112.5 (C-8a), 117.8 (C-7), 120.6 (C-5), 136.0 (C-6), 143.4 (C-4a), 160.5 (C-8), 165.4 (C-1), 167.3 (CO_2H); HRMS (TOF ESI+) calcd for $C_{11}H_{10}ClO_5$ $[M+H]^{\cdot+}$ 257.0211, found 257.0212.

3. Synthesis of the *N*-hydroxysuccinimidyl ester of hapten OTA*b* (OTA*b*-NHS ester)

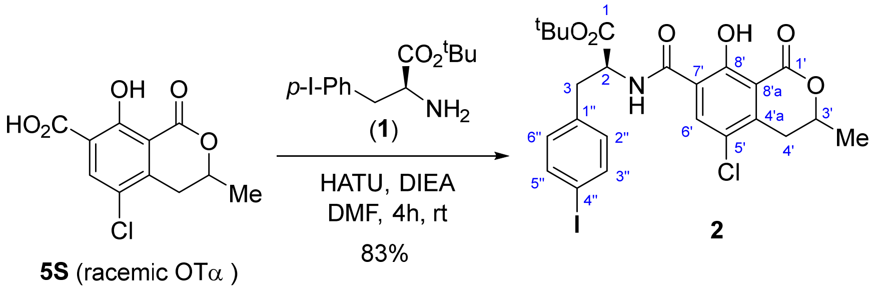

3.1. Preparation of *tert*-butyl (2*S*)-2-(5-chloro-8-hydroxy-3-methyl-1-oxoisochromane-7-carboxamido)-3-(4-iodophenyl)propanoate (**2**). A solution of HATU (125.5 mg, 0.33 mmol, 1.5 equiv) and DIEA (80 µL, 0.44 mmol, 2 equiv) in anhydrous DMF (1.5 mL) was added successively to a solution of acid **5S** (56.3 mg, 0.22 mmol) in anhydrous DMF (1.5 mL). The reaction mixture was stirred for 2 h at rt and then a solution of *tert*-butyl (*S*)-2-amino-3-(4-iodophenyl)propanoate (**1**)[3]

[3] (a) Hagmann, W. K.; Delaszlo, S. E.; Doherty, G.; Chang, L. L. & Yang, G. X. Preparation of heterocyclic amides with amino acids as cell adhesion inhibitors. Patent Nº WO2001012183A1 (Feb 22, 2001). (b) Yang, G. X.; Chang, L. L.; Truong, Q.; Doherty, G. A.; Magriotis, P. A.; de Laszlo, S. E.; Li, B.; MacCoss, M.; Kidambi, U.; Egger, L. A.; McCauley, E.; Van Riper, G.; Mumford, R. A.; Schmidt, J. A. & Hagmann, W. K. *N*-Tetrahydrofuroyl-(*L*)-phenylalanine derivatives as potent VLA-4 antagonists. *Bioorg Med Chem Lett. 12*, 1497-1500 (2002).

(153 mg, 0.44 mmol, 2 equiv) and DIEA (80 µL, 0.44 mmol, 2 equiv) in anhydrous DMF was added and stirred at rt for 4 h, after which the reaction was diluted with EtOAc and washed successively with aqueous solutions of HCl (1M), LiCl (1.5%), $NaHCO_3$ (5%) and brine, dried over anhydrous $MgSO_4$ and concentrated in the rotary evaporator. The obtained residue was purified by column chromatography, using hexane-EtOAc-AcOH mixtures (100:0:0.3, 90:10:0.3 and 80:20:0.3) as eluent, to obtain compound **2** (107 mg, 83%) as a yellowish oil (a 1:1 mixture of diastereoisomers). ^1H NMR ($CDCl_3$, 300 MHz) δ (ppm) 1.43 (two s, each 4.5H, CMe_3 of each diastereoisomer), 1.61 (two d, *J* = 6.4 Hz, each 1.5H, Me-3′ of each diastereoisomer), 2.84 and 2.89 (two dd, *J* = 17.4, 11.6 Hz, each 0.5H, H-4′ of each diastereoisomer), 3.07–3.24 (m, 2H, H_2-3), 3.30 (dd, *J* = 17.4, 3.5 Hz, 1H, H′-4′), 4.77 (m, 1H, H-3′), 4.94 (dt, *J* = 7.3, 6.0 Hz, 1H, H-2), 6.96 (br d, *J* = 7.9 Hz, 2H, H-2′′ and H-6′′), 7.56–7.62 (m, 2H, H-3′′ and H-5′′), 8.44 (s, 1H, H-6′), 8.57 (m, 1H, NH), 12.78 (br s, 1H, OH); ^{13}C NMR ($CDCl_3$, 75 MHz) δ (ppm) 20.7 (Me-3′), 28.0 (C*Me*$_3$), 32.3 (C-4′), 37.6 (C-3), 54.3 (C-2), 75.9 (C-3′), 82.6 (*C*Me$_3$), 92.4 (C-4′′), 110.0 (C-8′a), 120.7 (C-7′), 123.1 (C-5′), 131.5 (C-2′′ and C-6′′), 136.1 (C-1′′), 137.4 (C-3′′ and C-5′′), 138.9 (C-6′), 140.7 (C-4′a), 159.0 (C-8′), 162.1 (CONH), 169.7 (C-1′), 170.1 (C-1); HRMS (TOF ESI+) calcd for $C_{24}H_{26}ClINO_6$ $[M+H]^{\cdot+}$ 586.0488, found 586.0459.

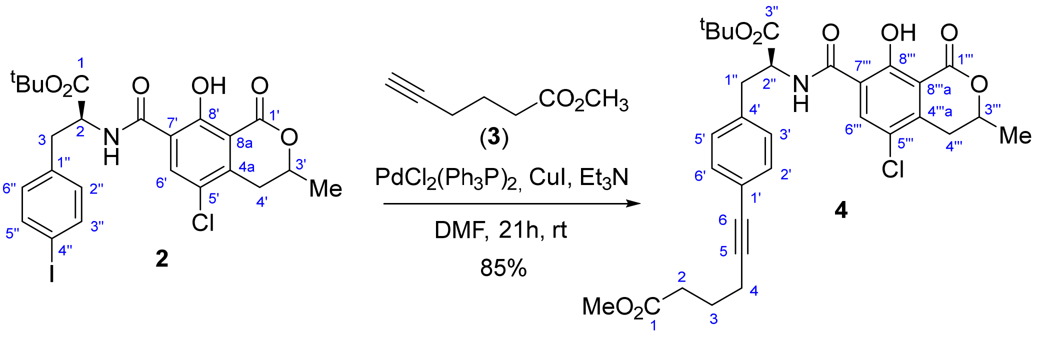

3.2. Preparation of methyl 6-(4-((2*S*)-3-(*tert*-butoxy)-2-(5-chloro-8-hydroxy-3-methyl-1-oxoisochromane-7-carboxamido)-3-oxopropyl)phenyl)hex-5-ynoate (**4**). A mixture of iodinated derivative **2** (36.5 mg, 0.062 mmol), methyl hex-5-ynoate (**3**, 27.4 mg, 0.217 mmol, 3.5 equiv), $PdCl_2(Ph_3P)_2$ (5.1 mg, 7.3 10^{-3} mmol , 0.12 equiv) and CuI (2.2 mg, 11.5·10^{-3} mmol, 0.18 equiv) was cooled to 0 °C and purged by repeated vacuum-nitrogen cycles. Next, anhydrous DMF (730 µL) and Et_3N (540 µL, 3.87 mmol, 62 equiv) were added and the mixture was purged again. The reaction mixture was stirred at rt for 21 h, after which it was diluted with EtOAc and washed successively with aqueous solutions of HCl (1M), LiCl (1.5%), $NaHCO_3$ (5%) and brine. The organic phase was dried over anhydrous $MgSO_4$ and concentrated under reduced pressure to give a residue which was purified by column chromatography on silica gel, using hexane-EtOAc-AcOH mixtures (80:20:0.3, 70:30:0.3 and 50:50:0.3) as an eluent, to obtain alkyne **4** (30.8 mg, 85%) as a yellowish oil (a 1:1 mixture of diastereoisomers) ^1H NMR ($CDCl_3$, 300 MHz) δ (ppm) 1.43 (br s, 9H, CMe_3), 1.60 (two d, *J*

= 6.3 Hz, each 1.5H, Me-3‴ of each diastereoisomer), 1.87–1.98 (m, 2H, H_2-3), 2.48 (t, *J* = 6.9 Hz, 2H, H_2-4), 2.51 (t, *J* = 7.3 Hz, 2H, H_2-2), 2.84 and 2.89 (two dd, *J* = 17.5, 11.5 Hz, each 0.5H, H-4‴ of each diastereoisomer), 3.19 (m, 2H, H_2-1″), 3.30 (dd, *J* = 17.4, 3.5 Hz, 1H, H′-4‴), 3.68 (s, 3H, CH_3O), 4.71–4.82 (m, 1H, H-3‴), 4.96 (dt, *J* = 7.3, 6.0 Hz, 1H, H-2″), 7.14 (d, *J* = 7.9 Hz, 2H, H-3′ and H-5′), 7.30 (dd, *J* = 8.2, 1.4 Hz, 2H, H-2′ and H-6′), 8.45 (s, 1H, H-6‴), 8.56 (m, 1H, NH), 12.76 (s, 1H, OH); ^{13}C NMR ($CDCl_3$, 75 MHz) δ (ppm) 18.9 (C-4), 20.7 (Me-3‴), 23.9 (C-3), 28.0 (C*Me*$_3$), 32.3 (C-4‴), 32.9 (C-2), 38.0 (C-1″), 51.6 (CH_3O), 54.5 (C-2″), 75.8 (C-3‴), 81.3 (C-6), 82.5 (*C*Me$_3$), 88.8 (C-5), 110.1 (C-8‴a), 120.8 (C-7‴), 122.3 (C-1′), 123.1 (C-5‴), 129.4 (C3′ and C-5′), 131.5 (C-2′ and C-6′), 136.0 (C-4′), 139.0 (C-6‴), 140.6 (C-4‴a), 159.1 (C-8‴), 162.1 (CONH), 169.7 (C-1‴), 170.2 (C-3″), 173.6 (C-1); HRMS (TOF ESI+) calcd for $C_{31}H_{35}ClNO_8$ $[M+H]^{\cdot+}$ 584.2046, found 584.2029.

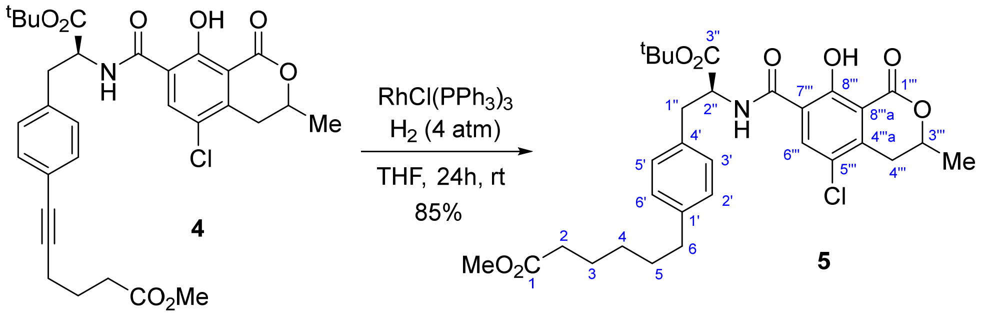

3.3. Preparation of methyl 6-(4-((2*S*)-3-(*tert*-butoxy)-2-(5-chloro-8-hydroxy-3-methyl-1-oxoisochromane-7-carboxamido)-3-oxopropyl)phenyl)hexanoate (**5**). A solution of alkyne **4** (17.6 mg, 0.03 mmol) and $RhCl(PPh_3)_3$ (4.2 mg, 4.5 10^{-3} mmol, 0.15 equiv) in anhydrous THF (1.3 mL) contained in a Büchi 'Tiny Clave' reactor was purged with hydrogen. The hydrogen pressure is adjusted to 4 atm and maintained with stirring at rt for 24 h. The reactor was then vented and the reaction mixture concentrated to dryness under vacuum to obtain a residue which was purified by column chromatography, using hexane-EtOAc mixtures (90:10, 80:20 and 60:40) as eluent, to obtain compound **5** (15.0 mg, 85%) as a yellowish oil (a 1:1 mixture of diastereoisomers) ^{1}H NMR ($CDCl_3$, 300 MHz) δ (ppm) 1.31–1.38 (m, 2H, H_2-4), 1.42 (br s, 9H, CMe$_3$), 1.54–1.71 (m, 4H, H_2-3, H_2-5), 1.60 (two d, *J* = 6.2 Hz, each 1.5H, Me-3‴ of each diastereoisomer), 2.30 (t, *J* = 7.5 Hz, 2H, H_2-2), 2.57 (t, *J* = 7.6 Hz, 2H, H_2-6), 2.83 and 2.88 (two dd, *J* = 17.4, 11.6 Hz, each 0.5H, H-4‴ of each diastereoisomer), 3.17 (d, *J* = 6.0 Hz, 2H, H_2-1″), 3.29 (dd, *J* = 17.4, 3.5 Hz, 1H, H′-4‴), 3.66 (s, 3H, OMe), 4.69–4.82 (m, 1H, H-3‴), 4.94 (dt, *J* = 7.3, 6.0 Hz, 1H, H-2″), 7.03–7.16 (m, 4H, H-2′, H-3′, H-5′ and H-6′), 8.46 (s, 1H, H-6‴), 8.54 (m, 1H, NH), 12.72 and 12.73 (two s, each 0.5H, OH of each diastereoisomer); ^{13}C NMR ($CDCl_3$, 75 MHz) δ (ppm) 20.7 (Me-3‴), 24.8 (C-3), 28.0 (C*Me*$_3$), 28.7 (C-4), 31.0 (C-5), 32.3 (C-4‴), 34.0 (C-2), 35.3 (C-6), 37.8 (C-1″), 51.4 (OMe), 54.7 (C-2″), 75.8 (C-3‴), 82.2 (*C*Me$_3$), 110.0 (C-8‴a), 121.0 (C-7‴), 123.0 (C-5‴), 128.4 (C-3′ and C-5′), 129.4 (C-2′ and C-6′),

133.5 (C-4’), 139.0 (C-6’’’), 140.5 (C-4’’’a), 141.1 (C-1’), 159.1 (C-8’’’), 162.1 (CONH), 169.8 (C-1’’’), 170.5 (C-3’’), 174.2 (C-1); HRMS (TOF ESI+) calcd for $C_{31}H_{39}ClNO_8$ $[M+H]^{·+}$ 588.2359, found 588.2358.

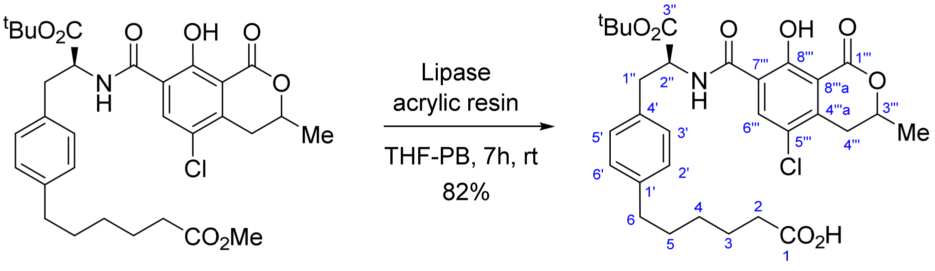

5 **6**

3.4. **Preparation of 6-(4-((2*S*)-3-(*tert*-butoxy)-2-(5-chloro-8-hydroxy-3-methyl-1-oxoisochromane-7-carboxamido)-3-oxopropyl)phenyl)hexanoic acid** (**6**). A suspension of methyl ester **5** (13.4 mg, 0.023 mmol) and lipase from *Candida antarctica* immobilized on acrylic resin (Novozyme 435, 26.4 mg) in THF (160 µL) in a phosphate buffer solution with pH = 7.4 (630 µL) was stirred at rt for 7 h. The mixture was filtered and the resin was washed with water and EtOAc. The aqueous phase was acidified with a 1M aqueous solution of HCl until pH 2-3 and extracted with EtOAc. The combined organic phases were washed with brine, dried over anhydrous $MgSO_4$ and concentrated to dryness under reduced pressure to obtain an oily residue which was purified by column chromatography on silica gel, using $CHCl_3$-MeOH mixtures (100:0, 95:5 and 90:10) as eluent, to give recovered methyl ester **5** (1.1 mg, 8.2%), followed by acid **6** (9.9 mg, 82% based on recovered starting material) as a yellowish oil (a 1:1 mixture of diastereoisomers). ^{1}H NMR ($CDCl_3$, 300 MHz) δ (ppm) 1.31–1.37 (m, 2H, H_2-4), 1.43 (s, 9H, CMe_3), 1.54–1.70 (m, 4H, H_2-3, H_2–5), 1.60 (br d, *J* = 6.2 Hz, , Me-3’’’), 2.32 (t, *J* = 7.3 Hz, 2H, H_2-2), 2.57 (t, *J* = 7.5 Hz, 2H, H_2-6), 2.78–2.92 (m, 1H, H-4’’’), 3.17 (m, 2H, H_2-1’’), 3.29 (dd, *J* = 17.4, 3.3 Hz, 1H, H’-4’’’), 4.68–4.82 (m, 1H, H-3’’’), 4.88–5.00 (m, 1H, H-2’’), 7.02–7.16 (m, 4H, H-2’, H-3’, H-5’ and H-6’), 8.45 (s, 1H, H-6’’’), 8.56 (m, 1H, NH), 12.73 (br s, 1H, OH); ^{13}C NMR ($CDCl_3$, 125 MHz) δ (ppm) 20.7 (Me-3’’’), 24.5 (C-3), 28.0 (C*Me*$_3$), 28.4 (C-4), 30.9 (C-5), 32.3 (C-4’’’), 33.8 (C-2), 35.2 (C-6), 37.8 (C-1’’), 54.7 (C-2’’), 75.9 (C-3’’’), 82.3 (*C*Me$_3$), 110.0 (C-8’’’a), 121.0 (C-7’’’), 123.0 (C-5’’’), 128.4 (C-3’ and C-5’), 129.4 (C-2’ and C-6’), 133.5 (C-4’), 139.0 (C-6’’’), 140.6 (C-4’’’a), 141.1 (C-1’), 159.1 (C-8’’’), 162.2 (CONH), 169.8 (C-1’’’), 170.5 (C-3’’), 179.3 (CO_2H); HRMS (TOF ESI+) calcd for $C_{30}H_{37}ClNO_8$ $[M+H]^{·+}$ 574.2202, found 574.2220.

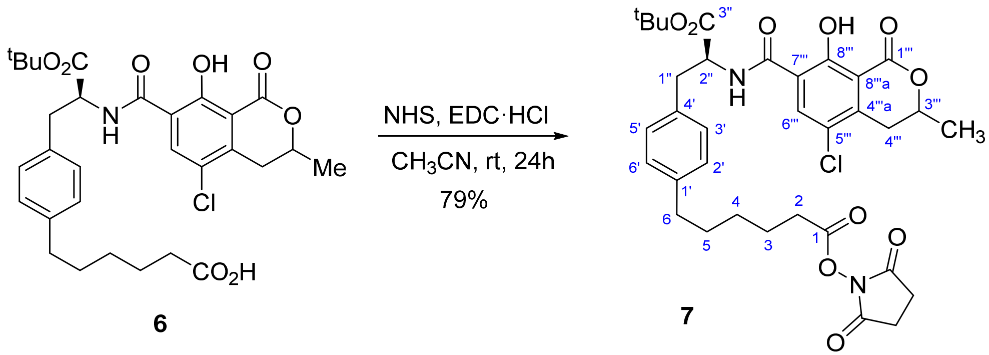

3.5. Preparation of 2,5-dioxopyrrolidin-1-yl 6-(4-((2*S*)-4-(*tert*-butoxy)-2-(5-chloro-8-hydroxy-3-methyl-1-oxoisochromane-7-carboxamido)-3,4-dioxobutyl)phenyl)hexanoate (**7**). A solution of acid **6** (10.2 mg, 0.018 mmol), NHS (2.2 mg, 0.019 mmol, 1.1 equiv) and EDC·HCl (3.7 mg, 0.019 mmol, 1.1 equiv) in anhydrous CH_3CN (1.0 mL) was stirred at rt for 24 h under nitrogen atmosphere. After this time, the reaction mixture was diluted with EtOAc and washed successively with water, a 5% aqueous solution of $NaHCO_3$ and brine. After evaporating to dryness, the residue obtained was chromatographed through a small column of silica gel, using CH_2Cl_2 as eluent, to obtain *N*-hydroxysuccinimidyl ester **7** (9.6 mg, 79%) as a yellowish oil (a 1:1 mixture of diastereoisomers). ^{1}H NMR ($CDCl_3$, 500 MHz) δ (ppm) 1.42 (br s, 9H, CMe$_3$), 1.43–1.49 (m, 2H, H$_2$-4), 1.58–1.67 (m, 2H, H$_2$-5), 1.60 (two d, each 1.5H, *J* = 6.4 Hz, Me-3’’’ of each diastereoisomer), 1.77 (quint, *J* = 7.6 Hz, 2H, H$_2$-3), 2.59 (br t, 4H, *J* = 7.5 Hz, H$_2$-2 and H$_2$-6), 2.79–2.90 (m, 5H, H-4’’’, CO*CH$_2$CH$_2$*CO), 3.11–3.22 (m, 2H, H$_2$-1’’), 3.29 (dd, *J* = 17.4, 3.5 Hz, 1H, H-4’’’), 4.69–4.81 (m, 1H, H-3’’’), 4.89–4.97 (m, 1H, H-2’’), 7.06–7.16 (m, 4H, H-2’, H-3’, H-5’ and H-6’), 8.45 (s, 1H, H-6’’’), 8.54 (m, 1H, NH), 12.73 (br s, 1H, OH); ^{13}C NMR ($CDCl_3$, 125 MHz) δ (ppm) 20.7 (Me-3’’’), 24.4 (C-3), 25.6 (CO*CH$_2$CH$_2$*CO), 28.0 (C*Me*$_3$), 28.4 (C-4), 30.8 (C-5), 30.9 (C-2), 32.3 (C-4’’’), 35.2 (C-6), 37.8 (C-1’’), 54.7 (C-2’’), 75.9 (C-3’’’), 82.2 (*C*Me$_3$), 110.0 (C-8’’’a), 121.0 (C-7’’’), 123.0 (C-5’’’), 128.4 (C-3’ and C-5’), 129.5 (C-2’ and C-6’), 133.6 (C-4’), 139.0 (C-6’’’), 140.5 (C-4’’’a), 140.9 (C-1’), 159.1 (C-8’’’), 162.1 (CONH), 168.6 (C-1), 169.1 (*CO*CH$_2$CH$_2$*CO*), 169.8 (C-1’’’), 170.5 (C-3’’); HRMS (TOF ESI+) calcd for $C_{34}H_{40}ClN_2O_{10}$ $[M+H]^{\cdot+}$ 671.2366, found 671.2349.

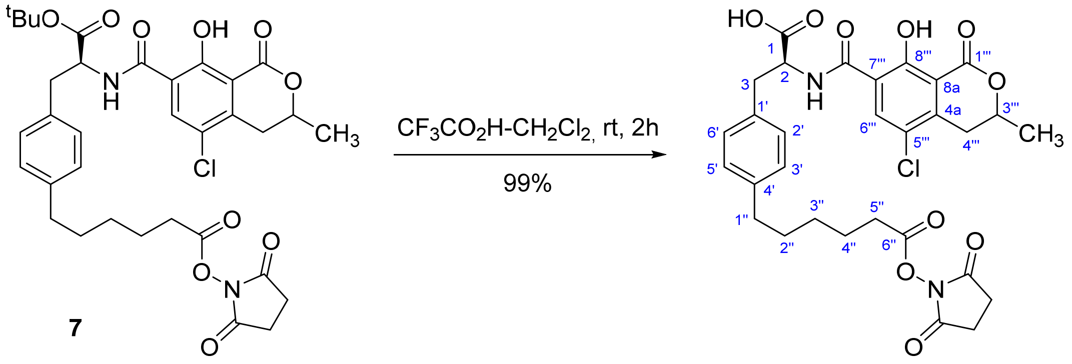

7 → **OTA*b*-NHS** ester

3.6. Preparation of (3*S*)-3-(5-chloro-8-hydroxy-3-methyl-1-oxoisochromane-7-carboxamido)-4-(4-(6-((2,5-dioxopyrrolidin-1-yl)oxy)-6-oxohexyl)phenyl)-2-oxobutanoic acid (OTA*b*-NHS ester). Trifluoroacetic acid (370 µL, 4.8 mmol) was dropwise added to a solution of *tert*-butyl ester **7** (9.0 mg, 0.013 mmol) in anhydrous CH_2Cl_2 (760 µL) and the resulting mixture was stirred for 2 h at rt. After this time, the reaction mixture was concentrated to dryness under reduced pressure to give OTA*b*-NHS ester (7.9 mg, nearly quantitative) as a resinous and brownish material (a 1:1 mixture of diastereoisomers). ^{1}H NMR ($CDCl_3$, 500 MHz) δ (ppm) 1.42 (m, 2H, H_2-3''), 1.57–1.68 (m, 2H, H_2-2''), 1.60 (two d, each 1.5H, *J* = 6.4 Hz, Me-3'''), 1.75 (m, 2H, H_2-4''), 2.59 (m, 4H, H_2-1'' and H_2-5''), 2.78–2.91 (m, 5H, H-4''', CO*CH₂CH₂*CO), 3.16–3.34 (m, 3H, H_2-3 and H'-4'''), 4.77 (m, 1H, H-3'''), 5.00–5.04 (m, 1H, H-2), 7.06–7.18 (m, 4H, H-2', H-3', H-5' and H-6'), 8.42 (m, 1H, H-6'''), 8.51 and 8.58 (two m, each 0.5H, NH of each diastereoisomer); HRMS (TOF ESI+) calcd for $C_{30}H_{32}ClN_2O_{10}$ $[M+H]^{·+}$ 615.1740, found 615.1732.

4. Synthesis of the *N*-hydroxysuccinimidyl ester of hapten OTA*d* (OTA*d*-NHS ester)

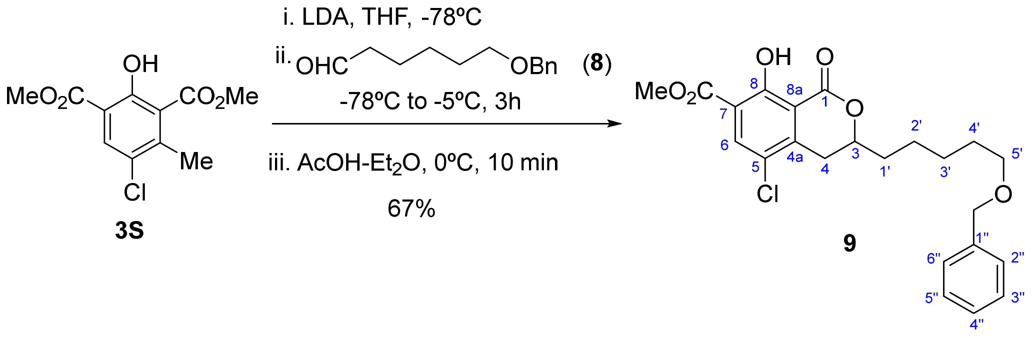

3S → **9**

4.1. Preparation of methyl 3-(5-(benzyloxy)pentyl)-5-chloro-8-hydroxy-1-oxoisochromane-7-carboxylate (9). A solution of diester **3S** (117.0 mg, 0.45 mmol) in anhydrous THF (280 µL) was added dropwise to a solution of LDA in THF [generated from diisopropylamine (185 µL, 1.25 mmol, 2.75 equiv) and 1.6 M BuLi in hexane (710 µL, 1.14 mmol, 2.5 equiv) in THF (1.4 mL)] at –78 °C under nitrogen. The mixture was maintained at the same temperature for 30 min and then a solution of

aldehyde **8**[4] (150 mg, 0.73 mmol, 1.6 equiv) in anhydrous THF (150 µL) was added. The reaction mixture was stirred at –78 °C for 15 min and then allowed to warm slowly to 5 °C (about 3 hours). At the end of this time, the reaction mixture was treated with a 1:2 mixture of AcOH-Et_2O and stirred for 10 minutes at 0 °C, diluted with EtOAc and washed with water and brine and dried over anhydrous $MgSO_4$. Chromatographic purification of the residue left after evaporation of the solvent under reduced pressure, using hexane-EtOAc-AcOH mixtures (100:0:0.3, 95:5:0.3 and 85:15:0.3) as eluent, afforded compound **9** (109.0 mg, 67%) as a yellowish oil. ^{1}H NMR ($CDCl_3$, 300 MHz) δ (ppm) 1.30–1.93 (m, 8H, H_2-1′, H_2-2′, H_2-3′ and H_2-4′), 2.77 (dd, *J* = 17.3, 11.7 Hz, 1H, H-4), 3.16 (dd, *J* = 17.3, 3.4 Hz, 1H, H′-4), 3.43 (t, *J* = 6.3 Hz, 2H, H_2-5′), 3.89 (s, 3H, OMe), 4.44 (s, 2H, O*CH₂*Ph), 4.45–4.58 (m, 1H, H-3), 6.98–7.46 (m, 5H, Ph), 8.05 (s, 1H, H-6), 12.14 (s, 1H, OH); ^{13}C NMR ($CDCl_3$, 75 MHz) δ (ppm) 24.5 (C-2′), 25.9 (C-3′), 29.5 (C-4′), 30.9 (C-4), 34.6 (C-1′), 52.5 (OMe), 70.0 (C-5′), 72.8 (O*CH*$_2$Ph), 78.5 (C-3), 111.4 (C-8a), 118.2 (C-7), 121.7 (C-5), 127.5 (C-4′′), 127.6 (C-2′′ and C-6′′), 128.3 (C-3′′ and C-5′′), 137.9 (C-6), 138.5 (C-1′′), 142.3 (C-4a), 161.0 (C-8), 165.0 (C-1), 167.9 (CO_2); HRMS (TOF ESI+) calcd for $C_{23}H_{26}ClO_6$ $[M+H]^{\cdot+}$ 433.1412, found 433.1415.

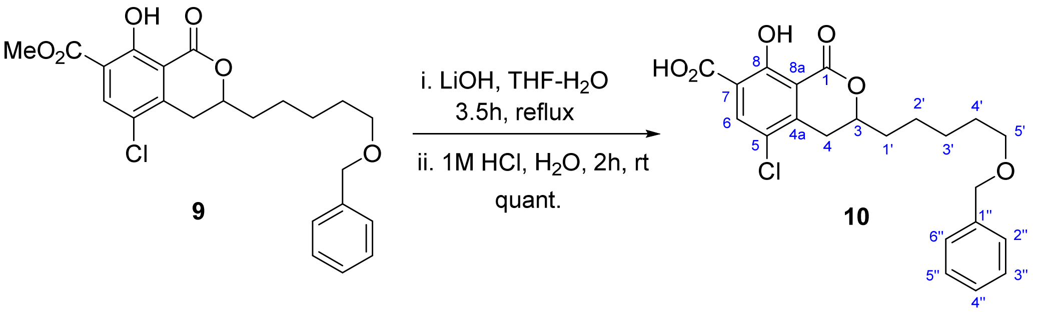

4.2. Preparation of 3-(5-(benzyloxy)pentyl)-5-chloro-8-hydroxy-1-oxoisochromane-7-carboxylic acid (10). A solution of LiOH·H_2O (135.0 mg, 3.21 mmol, 10 equiv) in water (1.40 mL) was added to a solution of dihydroisocoumarin **9** (139.0 mg, 0.32 mmol) in anhydrous THF (1.30 mL) and the mixture was heated at reflux for 3.5 h. The reaction mixture was then cooled in an ice bath and treated with a 1M aqueous solution of HCl (5.80 mL, 5.80 mmol, 18 equiv), stirred at 0 °C for 2 h, diluted with water and extracted with EtOAc. The combined organic phases were washed with brine, dried over anhydrous $MgSO_4$ and concentrated to dryness under reduced pressure to obtain acid **10** (134.5 mg, 100%) as a yellowish semi-solid, whose ^{1}H NMR showed to have a sufficiently high purity to be used in the next stage without further purification. ^{1}H NMR (MeOD, 300 MHz) δ (ppm) 1.42–1.91 (m, 8H, H_2-1′, H_2-2′, H_2-3′ and H_2-4′), 2.87 (dd, *J* = 17.3, 11.7 Hz, 1H, H-4), 3.21-3.29 (dd, *J* = 17.3, 3.0 Hz, H′-4), 3.52 (t, *J* = 6.4 Hz, 2H, H_2-5′), 4.50 (s, 2H, O*CH*$_2$Ph), 4.56 (m, 1H, H-3), 7.21–7.37 (m, 5H,

[4] Koyanagi, T.; Leriche, G.; Onofrei, D.; Holland, G. P.; Mayer, M. & Yang, J. *Angew. Chem. Int. Ed*. 55, 1890–1893 (2016).

Ph), 8.12 (s, 1H, H-6); ^{13}C NMR (MeOD, 75 MHz) δ (ppm) 25.8 (C-2'), 27.2 (C-3'), 30.7 (C-4'), 32.5 (C-4), 35.7 (C-1'), 71.4 (C-5'), 74.0 (O*CH₂*Ph), 79.7 (C-3), 114.0 (C-8a), 118.6 (C-7), 122.8 (C-5), 128.8 (C-4''), 129.0 (C-2'' and C-6''), 129.5 (C-3'' and C-5''), 138.2 (C-6), 140.0 (C-1''), 145.4 (C-4a), 163.0 (C-8), 167.5 (C-1), 169.8 (CO_2H); HRMS (TOF ESI+) calcd for $C_{22}H_{24}ClO_6$ $[M+H]^{\cdot+}$ 419.1256, found 419.1251.

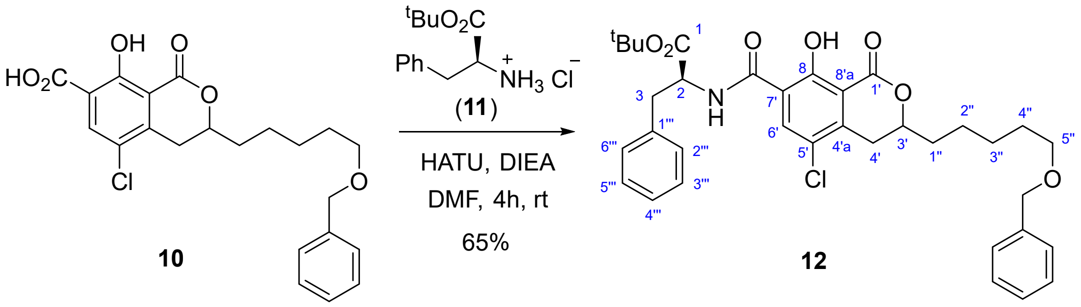

4.3. Preparation of *tert*-butyl (3-(5-(benzyloxy)pentyl)-5-chloro-8-hydroxy-1-oxoisochromane-7-carbonyl)-L-phenylalaninate (12). A solution of HATU (123.5 mg, 0.33 mmol, 1.5 equiv) in anhydrous DMF (1.5 mL) and DIEA (80 µL, 0.44 mmol, 2 equiv) were added to a solution of acid **10** (91.0 mg, 0.22 mmol) in anhydrous DMF (1.5 mL) under nitrogen and the resulting mixture was stirred at rt for 2 h. Next, a solution of hydrochloride **11** (108 mg, 0.44 mmol, 2 equiv) and DIEA (80 µL, 0.44 mmol, 2 equiv) in anhydrous DMF (1.1 mL) was added and the mixture stirred at the same temperature for 4 h. The reaction mixture was diluted with EtOAc and washed successively with aqueous solutions of HCl (1M), LiCl (1.5%), $NaHCO_3$ (5%) and brine, dried over anhydrous $MgSO_4$ and concentrated to dryness under reduced pressure. The residue obtained was purified by chromatography on silica gel, using hexane-EtOAc-AcOH mixtures (100:0:0.3, 90:10:0.3, and 80:20:0.3) as eluent to obtain amide **12** (87 mg, 65%) as a yellowish oil (a 1:1 mixture of diastereoisomers). ^{1}H NMR ($CDCl_3$, 300 MHz) δ (ppm) 1.28–1.91 (m, 8H, H_2-1'', H_2-2'', H_2-3'', H_2-4''), 1.36 (s, 9H, CMe_3), 2.69–2.86 (two dd, J = 17.3, 11.7 Hz, each 0.5H, H-4' of each diastereoisomer), 3.08–3.23 (m, 3H, H'-4' and H_2-3), 3.43 (t, J = 6.3 Hz, 2H, H_2-5''), 4.44 (s, 2H, O*CH₂*Ph), 4.47–4.59 (m, 1H, H-3'), 4.86–4.96 (m, 1H, H-2), 7.04–7.32 (m, 10H, 2xPh), 8.39 (s, 1H, H-6'), 8.50 (br d, J = 5.3 Hz, 1H, NH), 12.66 (s, 1H, OH); ^{13}C NMR ($CDCl_3$, 75 MHz) δ (ppm) 24.5 (C-2''), 25.9 (C-3''), 27.9 (C*Me₃*), 29.5 (C-4''), 30.6 (C-4'), 34.6 (C-1''), 38.1 (C-3), 54.6 (C-2), 70.0 (C-5''), 72.9 (O*CH₂*Ph), 79.2 (C-3'), 82.2 (*C*Me₃), 110.2 (C-8'a), 120.8 (C-7'), 123.0 (C-5'), 126.9 (C-4'''), 127.5 (C-4 Ph), 127.6 (C-2 and C-6 Ph), 128.3 (C-2''', C-6''', C-3 and C-5 Ph), 129.5 (C-3''' and C-5'''), 136.3 (C-1'''), 138.5 (C-1 Ph), 138.9 (C-6'), 140.6 (C-4'a), 159.0 (C-8'), 162.1 (CONH), 169.7 (C-1'), 170.3 (C-1); HRMS (TOF ESI+) calcd for $C_{35}H_{41}ClNO_7$ $[M+H]^{\cdot+}$ 622.2566, found 622.2570.

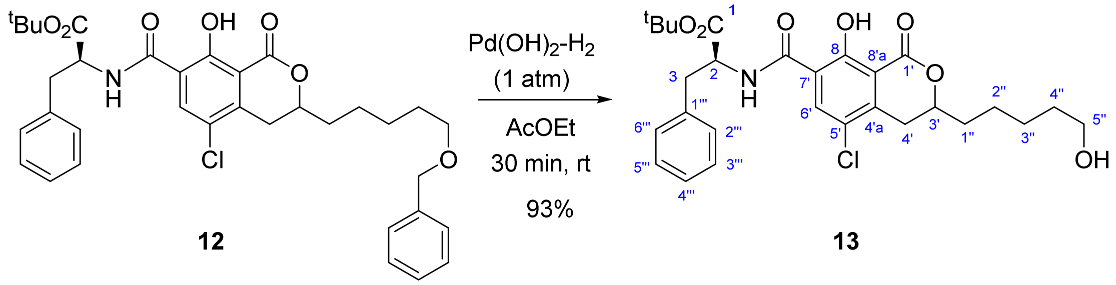

4.4. Preparation of *tert*-butyl (5-chloro-8-hydroxy-3-(5-hydroxypentyl)-1-oxoisochromane-7-carbonyl)-L-phenylalaninate (13). A suspension of $Pd(OH)_2$, (20% on carbon*,* 50% water wet, 44 mg, 0.06 mmol, 0.4 equiv) and benzyl ether **12** (85 mg, 0.14 mmol) in EtOAc (4.8 mL) was purged with repeated vacuum-hydrogen cycles and subsequently stirred vigorously under a pressure of H_2 of 1 atm (balloon) at rt for 30 min. The reaction mixture was filtered through a pad of silica gel using EtOAc for washing. The filtrate and washings were concentrated to dryness to provide alcohol **13** (67.2 mg, 93%) as a yellowish oil (a 1:1 mixture of diastereoisomers). ^{1}H NMR ($CDCl_3$, 300 MHz) δ (ppm) 1.40–1.98 (m, 8H, H_2-1'', H_2-2'', H_2-3'', H_2-4''), 1.42 (s, 9H, CMe_3), 2.78–2.94 (two dd, *J* = 17.4, 11.8 Hz, 1H, H-4' of each diastereoisomer), 3.14–3.33 (m, 3H, H-4' and H_2-3), 3.67 (t, *J* = 6.4 Hz, 2H, H_2-5''), 4.51–4.69 (m, 1H, H-3'), 4.96 (m, 1H, H-2), 7.10–7.35 (m, 5H, Ph), 8.44 (s, 1H, H-6'), 8.50–8.63 (two d, *J* = 7.4 Hz, each 0.5H, NH of each diastereoisomer), 12.72 (two s, each 0.5H, OH of each diastereoisomer); ^{13}C NMR ($CDCl_3$, 75 MHz) δ (ppm) 24.5 (C-2''), 25.5 (C-3''), 27.9 (C*Me*$_3$), 30.6 (C-4'), 32.4 (C-4''), 34.6 (C-1''), 38.1 (C-3), 54.6 (C-2), 62.6 (C-5''), 79.3 (C-3'), 82.3 (*C*Me$_3$), 110.1 (C-8'a), 120.8 (C-7'), 123.1 (C-5'), 126.9 (C-4'''), 128.3 (C-2''' and C-6'''), 129.5 (C-3''' and C-5'''), 136.2 (C-1'''), 138.9 (C-6'), 140.6 (C-4'a), 159.0 (C-8'), 162.1 (CONH), 169.7 (C-1'), 170.4 (C-1); HRMS (TOF ESI+) calcd for $C_{24}H_{27}ClNO_7$ $[M–C_4H_9]^{+}$ 476.1471, found 476.1451.

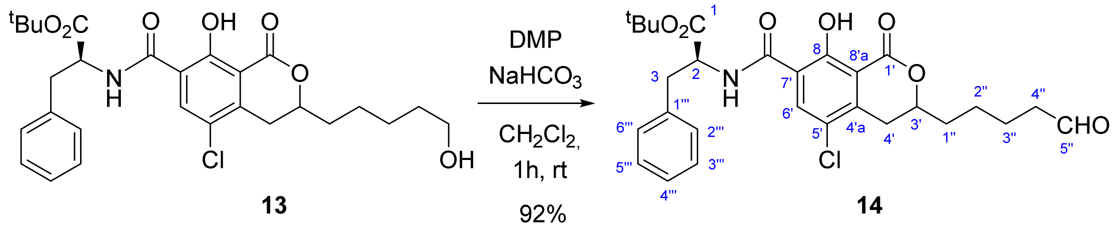

4.5. Preparation of tert-butyl (5-chloro-8-hydroxy-1-oxo-3-(5-oxopentyl)isochromane-7-carbonyl)-L-phenylalaninate (14). A mixture of Dess-Martin periodinane (DMP) (78.0 mg, 0.18 mmol, 1.5 equiv) and $NaHCO_3$ (82 mg, 0.98 mmol, 8.0 equiv) was added to a solution of alcohol **13** (65.0 mg, 0.12 mmol) in CH_2Cl_2 (4 mL) cooled at 0 °C. The resulting mixture was stirred at 0 °C for 10 min and then at rt for 1 h. After completion of the reaction, the mixture was diluted with EtOAc and washed successively with aqueous solutions of $Na_2S_2O_3$ (10%), saturated $NaHCO_3$ and brine, dried over anhydrous $MgSO_4$ and concentrated to dryness at reduced pressure to give aldehyde **14** (59.6

mg, 92%) as a yellowish oil (a 1:1 mixture of diastereoisomers). ^{1}H NMR ($CDCl_3$, 300 MHz) δ (ppm) 1.42 (s, 9H, CMe$_3$), 1.48–1.99 (m, 6H, H_2-1'', H_2-2'' and H_2-3''), 2.52 (dt, *J* = 7.0, 1.4 Hz, 2H, H_2-4''), 2.87 (two dd, *J* = 17.3, 11.7 Hz, each 0.5H, H-4' of each diastereoisomer), 3.14–3.31 (m, 3H, H'-4' and H_2-3), 4.60 (ddt, *J* = 11.7, 7.7, 4.0 Hz, 1H, H-3'), 4.92–5.01 (dt, J = 7.3, 6.1 Hz, 1H, H-2), 7.16–7.32 (m, 5H, Ph), 8.45 (s, 1H, H-6'), 8.56 (two d, *J* = 7.3 Hz, each 0.5H, NH of each diastereoisomer), 9.80 (t, *J* = 1.4 Hz, 1H, CHO), 12.69 (s, 1H, OH); ^{13}C NMR ($CDCl_3$, 75 MHz) δ (ppm) 21.6 (C-2''), 24.3 (C-3''), 27.9 (C*Me*$_3$), 30.6 (C-4'), 34.5 (C-1''), 38.1 (C-3), 43.6 (C-4''), 54.6 (C-2), 79.0 (C-3'), 82.3 (*C*Me$_3$), 110.1 (C-8'a), 120.9 (C-7'), 123.1 (C-5'), 126.9 (C-4'''), 128.3 (C-2''' and C-6'''), 129.5 (C-3''' and C-5'''), 136.3 (C-1'''), 139.0 (C-6'), 140.5 (C-4'a), 159.0 (C-8'), 162.3 (CONH), 169.6 (C-1'), 170.4 (C-1), 201.9 (C-5''); HRMS (TOF ESI+) calcd for $C_{28}H_{33}ClNO_7$ $[M+H]^{\cdot+}$ 530.1940, found 530.1920.

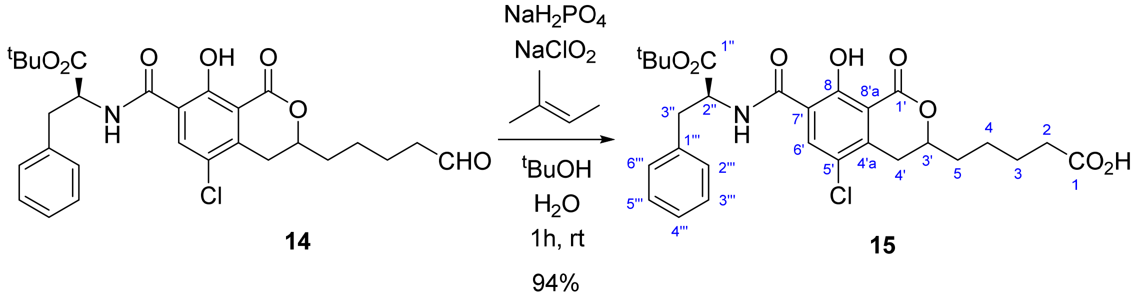

4.6. Preparation of 5-(7-(((*S*)-1-(*tert*-butoxy)-1-oxo-3-phenylpropan-2-yl)carbamoyl)-5-chloro-8-hydroxy-1-oxoisochroman-3-yl)pentanoic acid (15). A solution of NaH_2PO_4 (92.7 mg, 0.67 mmol, 6.0 equiv) and $NaClO_2$ (40.5 mg, 0.45 mmol, 4 equiv) in water (620 µL) was added to a solution of aldehyde **14** (59.6 mg, 0.11 mmol) and 2-methylbut-2-ene (175 µL, 1.57 mmol, 14 equiv) in *tert*-BuOH (1.5 mL) and the mixture was stirred at rt for 1 h. Then, a 1M aqueous solution of HCl (1.5 mL) was added and the mixture was stirred at rt for about 3 min, diluted with EtOAc, washed with brine and concentrated to dryness in vacuo to afford acid **15** (57.7 mg, 94%) as a yellowish oil (a 1:1 mixture of diastereoisomers). ^{1}H NMR ($CDCl_3$, 300 MHz) δ (ppm) 1.41 (s, 9H, CMe$_3$), 1.53–2.00 (m, 6H, H_2-3, H_2-4 and H_2-5), 2.42 (t, *J* = 7.10 Hz, 2H, H_2-2), 2.80–2.92 (two dd, *J* = 17.3, 11.9 Hz, each 0.5H, H-4' of each diastereoisomer), 3.13–3.31 (m, 3H, H'-4' and H_2-3''), 4.55–4.65 (m, 1H, H-3'), 4.97 (dt, *J* = 7.3, 6.1 Hz, 1H, H-2''), 7.11–7.31 (m, 5H, Ph), 8.44 (s, 1H, H-6'), 8.57–8.62 (m, 1H, NH), 12.71 (s, 1H, OH); ^{13}C NMR ($CDCl_3$, 75 MHz) δ (ppm) 24.2 (C-4), 27.9 (C*Me*$_3$), 29.6 (C-3), 30.6 (C-4'), 33.6 (C-2), 34.3 (C-5), 38.1 (C-3''), 54.6 (C-2''), 79.0 (C-3'), 82.4 (*C*Me$_3$), 110.1 (C-8'a), 120.7 (C-7'), 123.1 (C-5'), 126.9 (C-4'''), 128.3 (C-2''' and C-6'''), 129.5 (C-3''' and C-5'''), 136.2 (C-1'''), 139.0 (C-6'), 140.6 (C-4'a), 159.0 (C-8'), 162.2 (CONH), 169.6 (C-1'), 170.3 (C-1''), 178.6 (C-1); HRMS (TOF ESI+) calcd for $C_{28}H_{33}ClNO_8$ $[M+H]^{\cdot+}$ 546.1889, found 546.1867.

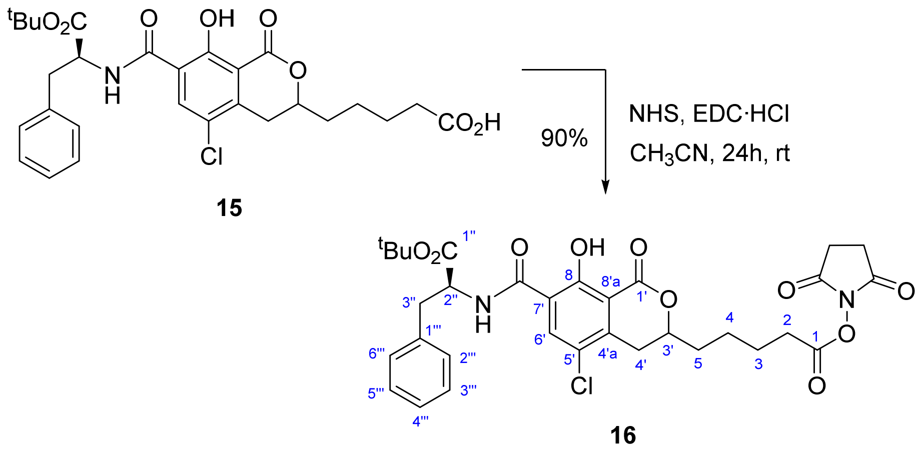

4.7. Preparation of 2,5-dioxopyrrolidin-1-yl 5-(7-(((*S*)-1-(*tert*-butoxy)-1-oxo-3-phenylpropan-2-yl)carbamoyl)-5-chloro-8-hydroxy-1-oxoisochroman-3-yl)pentanoate (**16**). A solution of acid **15** (31.8 mg, 0.058 mmol), NHS (7.3 mg, 0.064 mmol, 1.1 equiv) and EDC·HCl (13.4 mg, 0.07 mmol, 1.2 equiv) in dry CH_3CN (1.6 mL) was stirred at rt for 24 h under nitrogen, after which it was diluted with EtOAc and washed successively with water, 5% aqueous solution of $NaHCO_3$ and brine. After drying over anhydrous $MgSO_4$ and removing the solvent under reduced pressure, the *N*-hydroxysuccinimidyl ester **16** (33.0 mg, 90%) was obtained as an oil (a 1:1 mixture of diastereoisomers). ^{1}H NMR ($CDCl_3$, 300 MHz) δ (ppm) 1.41 (s, 9H, CMe_3), 1.50–1.91 (m, 6H, H_2-3, H_2-4 and H_2-5), 2.67 (t, *J* = 7.0 Hz, 2H, H_2-2), 2.83 (s, 4H, $COCH_2CH_2CO$), 2.88–2.94 (m, 1H, H-4'), 3.14–3.24 (m, 2H, H_2-3''), 3.27 (dd, *J* = 17.2, 3.3 Hz, H'-4'), 4.54–4.68 (m, 1H, H-3'), 4.90–5.01 (dt, *J* = 7.4, 6.3 Hz, 1H, H-2''), 7.12–7.34 (m, 5H, Ph), 8.44 (s, 1H, H-6'), 8.55 (two dd, *J* = 7.4 Hz, each 0.5H, NH of each diastereoisomer), 12.69 (s, 1H, OH); ^{13}C NMR ($CDCl_3$, 75 MHz) δ (ppm) 24.2 (C-4), 25.6 ($COCH_2CH_2CO$), 27.9 (CMe_3), 29.6 (C-3), 30.6 (C-2), 30.7 (C-4'), 34.1 (C-5), 38.1 (C-3''), 54.6 (C-2''), 78.9 (C-3'), 82.3 (CMe_3), 110.1 (C-8'a), 120.8 (C-7'), 123.1 (C-5'), 126.9 (C-4'''), 128.3 (C-2''' and C-6'''), 129.5 (C-3''' and C-5'''), 136.2 (C-1'''), 138.9 (C-6'), 140.5 (C-4'a), 159.0 (C-8'), 162.1 (CONH), 168.3 (C-1), 169.1 ($COCH_2CH_2CO$), 169.6 (C-1'), 170.3 (C-1''); HRMS (TOF ESI+) calcd for $C_{32}H_{36}ClN_2O_{10}$ $[M+H]^{\cdot+}$ 643.2053, found 643.2050.

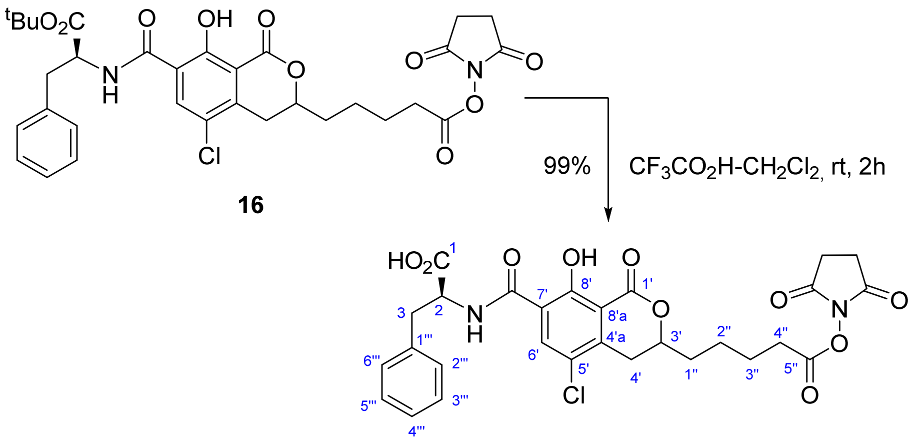

16

OTA*d*-NHS ester

4.8. Preparation of (5-chloro-3-(5-((2,5-dioxopyrrolidin-1-yl)oxy)-5-oxopentyl)-8-hydroxy-1-oxoisochromane-7-carbonyl)-L-phenylalanine (**OTA*d*-NHS ester**). A solution of the *tert*-butyl ester **16** (33.0 mg, 0.051 mmol) in CF_3CO_2H (1.5 mL) and anhydrous CH_2Cl_2 (2.3 mL) was stirred at rt for 2 h under nitrogen. The solvents were then removed to dryness under reduced pressure to obtain OTA*d*-NHS ester (29.8 mg, 99%) as a resinous, brownish-colored residue (a 1:1 mixture of diastereoisomers). ^1H NMR ($CDCl_3$, 300 MHz) δ (ppm) 1.54–1.93 (m, 6H, H_2-1'', H_2-2'' and H_2-3''), 2.68 (t, *J* = 6.69 Hz, 2H, H_2-4''), 2.85 (br s, 4H, COCH_2CH_2CO), 2.92 (m, 1H, H-4'), 3.17–3.43 (m, 3H, H_2-3 and H'-4'), 4.61 (m, 1H, H-3'), 5.03 (m, 1H, H-2), 7.18–7.33 (m, 5H, Ph), 8.39 (s, 1H, H-6'), 8.56 (br s, 1H, NH), 12.73 (br s, 1H, OH); HRMS (TOF ESI+) calcd for $C_{28}H_{28}ClN_2O_{10}$ $[M+H]^{\cdot+}$ 587.1427, found 587.1425.

5. Synthesis of the *N*-hydroxysuccinimidyl ester of hapten OTA*e* and OTA*f* (OTA*e*-NHS and OTA*f*-NHS esters)

5.1. Synthesis of hapten OTA*f*

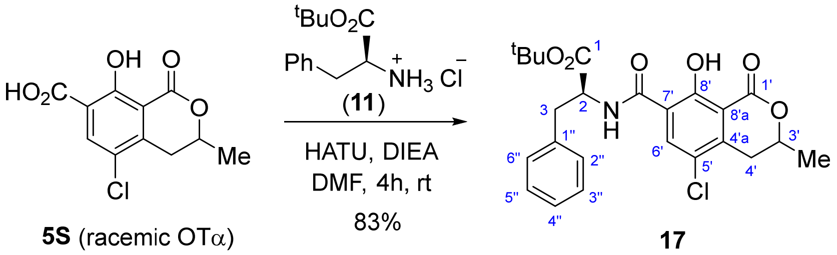

5S (racemic OTα)

17

5.1.1. Preparation of *tert*-butyl (5-chloro-8-hydroxy-3-methyl-1-oxoisochromane-7-carbonyl)-L-phenylalaninate (**17**). A solution of HATU (91.3 mg, 0.24 mmol, 1.5 equiv) and DIEA (60 µL, 0.32 mmol, 2 equiv) in anhydrous DMF (1.1 mL) was added to a solution of **5S** (40 mg, 0.16 mmol) in anhydrous DMF (1.1 mL) and the resulting mixture was stirred at rt for 2 h. Next, a solution of hydrochloride **11** (82.5 mg, 0.32 mmol, 2 equiv) and DIEA (60 µL, 0.324 mmol, 2 equiv) in anhydrous

DMF (1.1 mL) was added and the reaction mixture was stirred at rt for 4 h. The reaction mixture was diluted with EtOAc and washed successively with aqueous solutions of HCl (1M), LiCl (1.5%), $NaHCO_3$ (5%) and brine, dried over anhydrous $MgSO_4$ and concentrated to dryness in the rotary evaporator. The obtained residue was purified by chromatography on silica gel, using hexane-EtOAc-AcOH mixtures (100:0:0.3, 90:10:0.3 and 80:20:0.3) as eluent, to obtain compound **17** (59.5 mg, 83%) as a yellowish oil (a 1:1 mixture of diastereoisomers). ^{1}H NMR ($CDCl_3$, 300 MHz) δ (ppm) 1.46 (s, 9H, CMe_3), 1.62–1.64 and 1.63–1.65 (two d, *J* = 6.3, each 1.5H, Me-3' of each diastereoisomer), 2.84–2.89 and 2.90–2.95 (two dd, *J* = 17.7, 11.7 Hz, each 0.5H, H-4' of each diastereoisomer), 3.25 (m, 2H, H_2-3), 3.33 (dd, *J* = 17.3, 3.4 Hz, 1H, H'-4'), 4.80 (m, 1H, H-3'), 5.01 (dt, *J* = 7.4, 6.0 Hz, 1H, H-2), 7.20–7.38 (m, 5H, Ph), 8.49 (s, 1H, H-6'), 8.58–8.62 (two d, *J* = 6.9 Hz, each 0.5H, NH of each diastereoisomer), 12.77 (s, 1H, OH); ^{13}C NMR ($CDCl_3$, 75 MHz) δ (ppm) 20.6 (Me-3'), 27.9 (C*Me*$_3$), 32.2 (C-4'), 38.1 (C-3), 54.5 (C-2), 75.8 (C-3'), 82.3 (*C*Me$_3$), 110.0 (C-8'a), 120.8 (C-7'), 123.0 (C-5'), 126.9 (C-4''), 128.3 (C-2'' and C-6''), 129.5 (C-3'' and C-5''), 136.2 (C-1''), 138.9 (C-6'), 140.5 (C-4'a), 159.0 (C-8'), 162.1 (CONH), 169.7 (C-1'), 170.3 (C-1); HRMS (TOF ESI+) calcd for $C_{24}H_{27}ClNO_6$ $[M+H]^{\cdot+}$ 460.1521, found 460.1522.

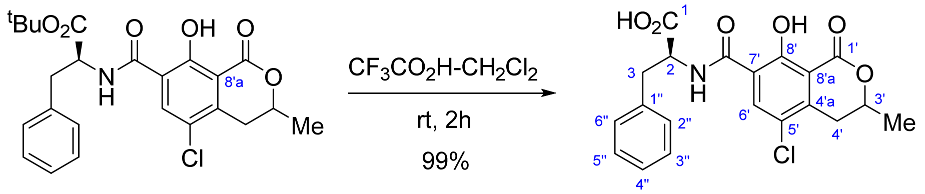

17 OTA*f*

5.1.2. Preparation of (5-chloro-8-hydroxy-3-methyl-1-oxoisochromane-7-carbonyl)-L-phenylalanine (Hapten OTA*f*). Trifluoroacetic acid (1.25 mL) was added to a solution of *tert*-butyl ester **17** (20.5 mg, 0.045 mmol) in anhydrous CH_2Cl_2 (2 mL). The reaction mixture was kept under stirring at rt for about 2 h and then concentrated to dryness under reduced pressure to give OTA*f* (17.6 mg, 99%) as a cream solid (a 1:1 mixture of diastereoisomers). ^{1}H NMR ($CDCl_3$, 300 MHz) δ (ppm) 1.60 (d, *J* = 6.2 Hz, 3H, Me-3'), 2.82 and 2.88 (two dd, *J* = 17.5, 11.5 Hz, each 0.5H, H-4' of each diastereoisomer), 3.16–3.40 (m, 3H, H_2-3 and H'-4'), 4.75 (m, 1H, H-3') 4.99–5.08 (m, 1 H, H-2) 7.10–7.37 (m, 5H, Ph) 8.42 (s, 1H, H-6'), 8.50 (two d, *J* = 6.8 Hz, each 0.5H, NH of each diastereoisomer), 12.74 (s, 1H, OH); ^{13}C NMR ($CDCl_3$, 75 MHz) δ (ppm) 20.6 (Me-3'), 32.2 (C-4'), 37.3 (C-3), 54.3 (C-2), 75.9 (C-3'), 110.0 (C-8'a), 120.2 (C-7'), 123.2 (C-5'), 127.3 (C-4''), 128.3 (C-2'' and C-6''), 129.3 (C-3'' and C-5''), 135.7 (C-1''), 138.9 (C-6'), 141.0 (C-4'a), 159.0 (C-8'), 163.1 (CONH), 169.7 (C-1'), 175.1 (C-1); HRMS (TOF ESI+) calcd for $C_{20}H_{19}ClNO_6$ $[M+H]^{\cdot+}$ 404.0895, found 404.0901.

5.2. Synthesis of hapten OTA*e*

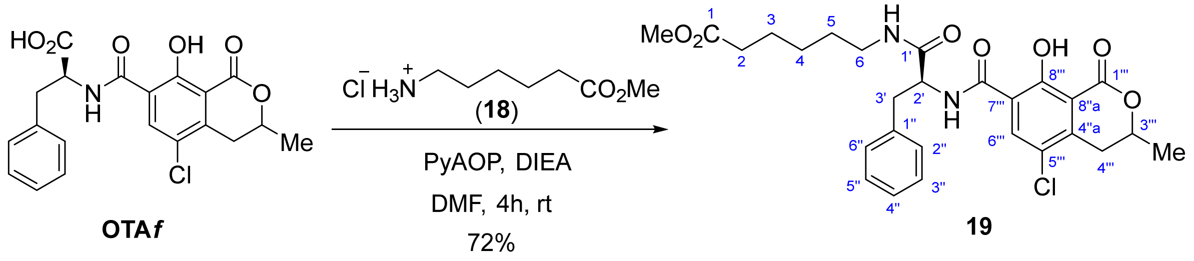

5.2.1. Preparation of methyl 6-((2*S*)-2-(5-chloro-8-hydroxy-3-methyl-1-oxoisochromane-7-carboxamido)-3-phenylpropanamido)hexanoate (**19**). DIEA (25 µL, 0.143 mmol, 3.25 equiv) was added to a solution of OTA*f* (18 mg, 0.044 mmol) and 6-methoxy-6-oxohexan-1-ammonium chloride (**18**, 9.4 mg, 0.052 mmol, 1.1 equiv) in anhydrous DMF (600 µL) under nitrogen. The resulting mixture was stirred for 10 min and then a solution of PyAOP (34 mg, 0.065 mmol, 1.5 equiv) in anhydrous DMF (600 µL) was added. After stirring at rt for 4 h, the mixture was diluted with EtOAc and washed with aqueous solutions of LiCl (1.5%), $NaHCO_3$ (5%) and brine and dried over anhydrous $MgSO_4$. Chromatography of the residue left after evaporation of the solvent, using mixtures of $CHCl_3$-MeOH (100:0 and 99:1) as eluent, gave amide **19** (16.8 mg, 72%). as a yellowish oil (a 1:1 mixture of diastereoisomers). ^{1}H NMR ($CDCl_3$, 300 MHz) δ (ppm) 1.11–1.28 (m, 2H, H_2-4), 1.31–1.44 (m, 2H, H_2-5), 1.49–1.63 (m, 2H, H_2-3), 1.61 (two d, *J* = 6.4 Hz, each 1.5H, Me-3''' of each diastereoisomer), 2.17–2.34 (t, *J* = 7.4 Hz, 2H, H_2-2), 2.86 (two dd, *J* = 17.5, 11.6 Hz, each 0.5H, H-4''' of each diastereoisomer), 3.04–3.37 (m, 5H, H_2-6, H_2-3' and H'-4'''), 3.66 (s, 3H, OMe), 4.62–4.89 (m, 2H, H-3''' and NH), 5.83 (m, 1H, H-2'), 7.12–7.40 (m, 5H, Ph), 8.41 (s, 1H, H-6'''), 8.59 (d, *J* = 7.4 Hz, 1H, NH), 12.81 (s, 1H, OH); ^{13}C NMR ($CDCl_3$, 75 MHz) δ (ppm) 20.7 (Me-3'''), 24.4 (C-3), 26.2 (C-4), 28.9 (C-5), 32.2 (C-4'''), 33.8 (C-2), 38.4 (C-3'), 39.2 (C-6), 51.5 (OMe), 55.7 (C-2'), 75.9 (C-3'''), 110.1 (C-8'a), 120.6 (C-7'''), 123.1 (C-5'''),127.0 (C-4''), 128.6 (C-2'' and C-6''), 129.3 (C-3'' and C-5''), 136.8 (C-1''), 138.8 (C-6'''), 140.8 (C-4'a), 159.0 (C-8'''), 162.7 (CONH), 169.7 (C-1'''), 170.4 (C-1'), 173.9 (C-1); HRMS (TOF ESI+) calcd for $C_{27}H_{32}ClN_2O_7$ $[M+H]^{+}$ 531.1893, found 531.1878.

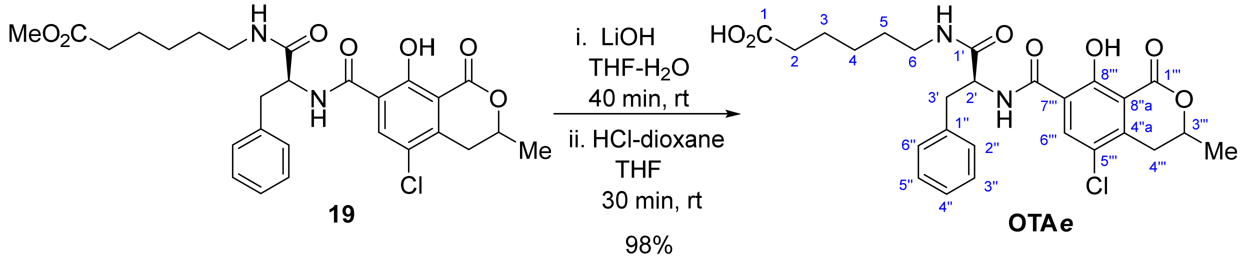

5.2.2. Preparation of 6-((2*S*)-2-(5-chloro-8-hydroxy-3-methyl-1-oxoisochromane-7-carboxamido)-3-phenylpropanamido)hexanoic acid (**Hapten OTA*e***). A solution of LiOH·H_2O (13 mg, 0.31 mmol, 10 equiv) in H_2O (700 µL) was added to a solution of methyl ester **19** (16.6 mg, 0.031 mmol) in THF (700

µL) and the mixture was stirred at rt for 40 min. The reaction mixture was then cooled in an ice bath and acidified with a 1M aqueous solution of $KHSO_4$ to a pH of approximately 2, diluted with EtOAc, washed with brine and dried over anhydrous $MgSO_4$. The residue obtained after evaporation of the solvent at reduced pressure was dissolved in anhydrous THF (1.2 mL) and treated with a drop of 4M HCl in dioxane. After stirring at rt for 30 min, the solvent was removed in vacuo to dryness to give hapten OTA*e* (15.7 mg, 98%) as a yellowish semi-solid (a 1: 1 mixture of diastereoisomers). ^{1}H NMR (DMSO-d_6, 500 MHz) δ (ppm) 1.18–1.28 (m, 2H, H_2-4), 1.30–1.40 (m, 2H, H_2-5), 1.42–1.52 (m, 2H, H_2-3), 1.46–1.47 (two d, *J* = 6.4 Hz, each 1.5H, Me-3''' of each diastereoisomer), 2.14–2.20 (t, *J* = 7.4 Hz, 2H, H_2-2), 2.88–3.08 (m, 6H, H-4''', H_2-3', H_2-6 and NH), 3.22 (dd, *J* = 17.2, 3.1 Hz, 1H, H'-4'''), 4.69–4.76 (m, 1H, H-3'''), 4.84 (m, 1H, H-2'), 7.15–7.28 (m, 5H, Ph), 8.08 and 8.09 (two s, each 0.5H, H-6''' of each diastereoisomer), 8.14 and 8.60 (two m, each 0.5H, NH of each diastereoisomer); ^{13}C NMR (DMSO-d_6, 125 MHz) δ (ppm) 20.0 (Me-3'''), 24.2 (C-3), 25.9 (C-4), 28.6 (C-5), 31.6 (C-4'''), 33.56 (C-2), 38.1 (C-3'), 38.4 (C-6), 54.5 (C-2'), 75.4 (C-3'''), 111.3 (C-8'''a), 120.2 (C-7'''), 121.4 (C-5'''), 126.4 (C-4''), 128.1 (C-2'' and C-6''), 129.2 (C-3'' and C5''), 136.0 (C-1''), 139.2 (C-6'''), 141.6 (C-4'''a), 158.4 (C-8'''), 162.5 (CONH), 169.9 (C-1'''), 169.9 (C-1'), 174.3 (C-1); HRMS (TOF ESI+) calcd for $C_{26}H_{30}ClN_2O_7$ $[M+H]^{+}$ 517.1736, found 517.1731.

5.3. Preparation of OTA*e*-NHS and OTA*f*-NHS esters

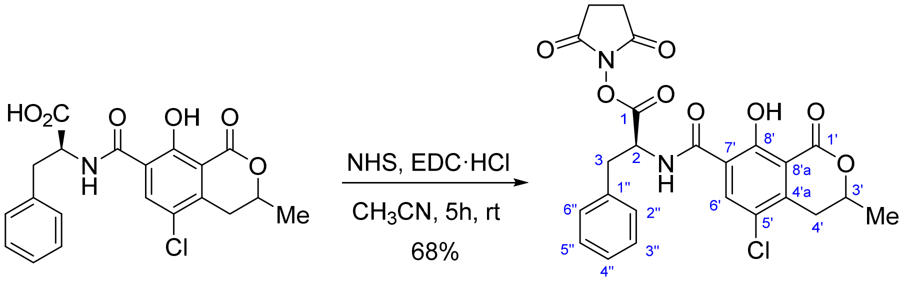

5.3.1. Preparation of 2,5-dioxopyrrolidin-1-yl (5-chloro-8-hydroxy-3-methyl-1-oxoisochromane-7-carbonyl)-L-phenylalaninate (**OTA*f*-NHS ester**). A solution of EDC·HCl (17.0 mg, 0.089 mmol, 1.1 equiv) in anhydrous CH_3CN (1 mL) was added dropwise to a solution of OTA*f* (30 mg, 0.074 mmol) and NHS (9.4 mg, 0.082 mmol, 1.1 equiv) in CH_3CN (1 mL) under nitrogen. The reaction mixture was stirred at rt for 6 h and them diluted with EtOAc and washed successively with water, a 5% aqueous solution of $NaHCO_3$ and brine. Evaporation of the solvent under reduced pressure gave the *N*-hydroxysuccinimidyl ester of hapten OTA*f* (25.2 mg, 68%) as a viscous oil (a 1:1 mixture of diastereoisomers). ^{1}H NMR ($CDCl_3$, 300 MHz) δ (ppm) 1.54–1.61 (d, *J* = 6.4 Hz, 3H, Me-3') 2.78–2.90 (m, 5H, H-4', $COCH_2CH_2CO$) 3.22–3.37 (m, 2H, H_2-3) 3.40–3.51 (m, 1H, H-4') 4.74 (m, 1H, H-3') 5.30–

5.42 (m, 1H, H-2), 7.21–7.39 (m, 5H, Ph) 8.37–8.48 (m, 2H, H-6′ and NH) 12.70 (s, 1H, OH); HRMS (TOF ESI+) calcd for $C_{20}H_{17}ClNO_5$ [M–$C_4H_4NO_3$]$^{·+}$ 386.0790, found 386.0785.

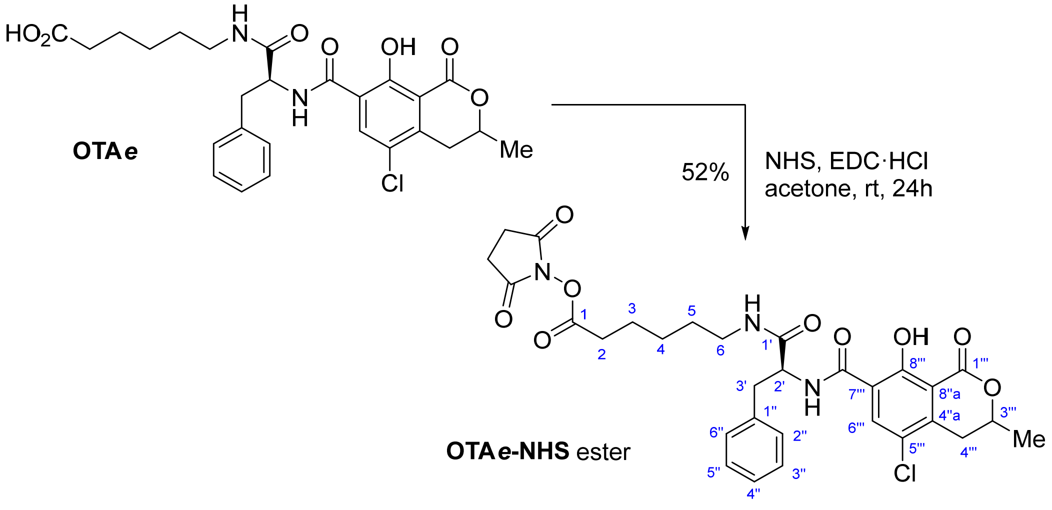

5.3.2. Preparation of 2,5-dioxopyrrolidin-1-yl 6-((2S)-2-(5-chloro-8-hydroxy-3-methyl-1-oxoisochromane-7-carboxamido)-3-phenylpropanamido)hexanoate (**OTA*e*-NHS ester**). A solution of EDC·HCl (7.0 mg, 0.037 mmol, 1.2 equiv) in anhydrous acetone (0.5 mL) was added to a solution of hapten OTA*e* (16 mg, 0.031 mmol) and NHS (3.9 mg, 0.034 mmol, 1.1 equiv) in anhydrous DMF (0.5 mL) under nitrogen. The mixture was stirred at rt for 24 h and then treated with a saturated aqueous solution of NH_4Cl, diluted with EtOAc and washed with aqueous solutions of LiCl (1.5%), $NaHCO_3$ (5%) and brine, and dried over anhydrous $MgSO_4$. The solvent was removed under reduced pressure and the residue purified by flash chromatography, using CH_2Cl_2-acetone mixtures (90:10, 85:15 and 80:20) as eluent, to give the *N*-hydroxysuccinimidyl ester of hapten OTA*e* (9.8 mg, 52%) as a yellowish oil (a 1:1 mixture of diastereoisomers). ^{1}H NMR (DMSO-d_6, 500 MHz) δ (ppm) 1.29–1.37 (m, 2H, H_2-4), 1.39–1.47 (m, 2H, H_2-5), 1.60 (d, *J* = 6.4 Hz, 3H, Me-3′′′), 1.64–1.78 (m, 2H, H_2-3), 2.56 (t, *J* = 6.8 Hz, 2H, H_2-2), 2.76–2.93 (m, 5H, H-4′′′, CO*CH₂CH₂*CO), 3.06–3.37 (m, 5H, H′-4′′′, H_2-3′ and H_2-6), 4.68–4.91 (m, 2H, H-3′′′ and NH), 6.04 (m, 1H, H-2′), 7.16–7.35 (m, 5H, Ph), 8.38 (s, 1H, H-6′′′), 8.60 (d, *J* = 7.0 Hz, 1H, NH), 12.79 (s, 1H, OH); HRMS (TOF ESI+) calcd for $C_{30}H_{33}N_3ClO_9$ [M+H]$^{·+}$ 614.1900, found 614.1892.

6. Preparation of bioconjugates

Each of the active esters of OTA haptens was dissolved in DMF and drop wise added to a protein solution in 50 mM carbonate buffer (pH 9.6). The conjugation reaction was performed with a 10% (v/v) DMF maximum content. After 2 h at room temperature under stirring, the protein conjugates were purified by size-exclusion chromatography using 100 mM phosphate buffer (pH 7.4) as eluent. BSA conjugates were filter sterilized and stored frozen at −20 °C. OVA conjugates

were diluted in elution buffer and kept at −20 °C in PBS (10 mM phosphate buffer, pH 7.4, with 140 mM NaCl) containing 0.01% thimerosal. HRP conjugates were diluted with PBS containing 1% (w/v) BSA and 0.01% thimerosal and stored at 4 °C.

7. MALDI mass spectrometry analysis of immunizing and assay bioconjugates

Sample preparation. 100 µL of each of the protein conjugate solutions (0.5–1 mg/mL) were dialyzed against milliQ water and then freeze-dried and lyophilized. The samples were dissolved in MilliQ H_2O to theoretical final concentration 1 µg/µL. Then, 1 µL of every sample solution was spotted onto the MALDI plate. After the droplets were air dried at room temperature, 1 µL of matrix (10 mg/mL sinapinic acid (Bruker) in 0.1% trifluoroacetic acid–CH_3CN/H_2O (7:3 v/v) was added and allowed to air-dry at room temperature.

Mass spectrometry analysis. The resulting mixtures were analyzed in a 5800 MALDI TOF/TOF (ABSciex) in positive linear mode (1500 shots every position) in a mass range of 15000–100000 *m/z*. Previously, the plate was calibrated with 1 µL of the TOF/TOF calibration mixture (ABSciex), in 13 positions. Every sample was calibrated by 'close external calibration' method with a BSA, OVA, or HRP spectrum acquired in a close position.

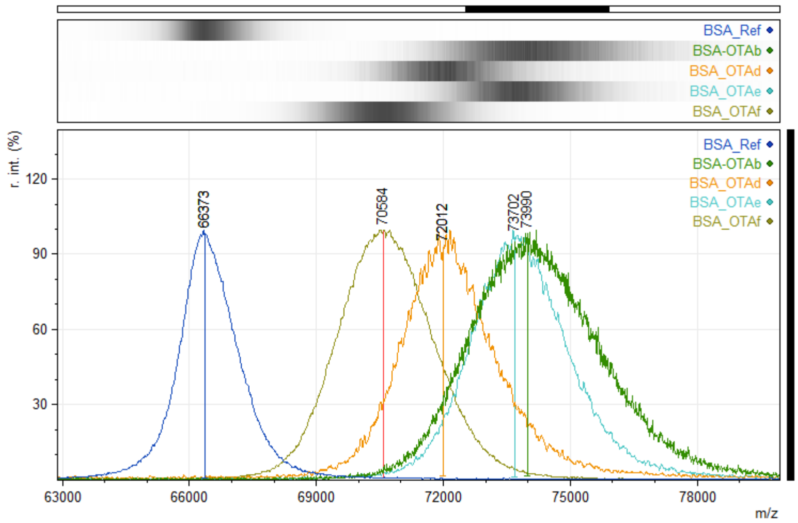

Figure S2. MALDI-TOF-MS spectra (singly charged ions) of BSA (blue) and the corresponding conjugates with haptens OTA*b* (green), OTA*d* (orange), OTA*e* (cyan), and OTA*f* (olive).

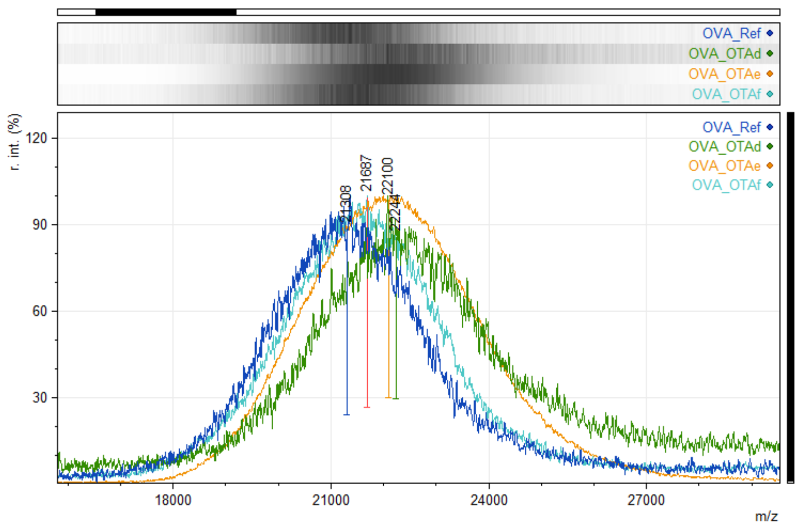

Figure S3. MALDI-TOF-MS spectra (doubly charged ions) of OVA (blue) and the corresponding conjugates with haptens OTA*d* (green), OTA*e* (orange) and OTA*f* (cyan).

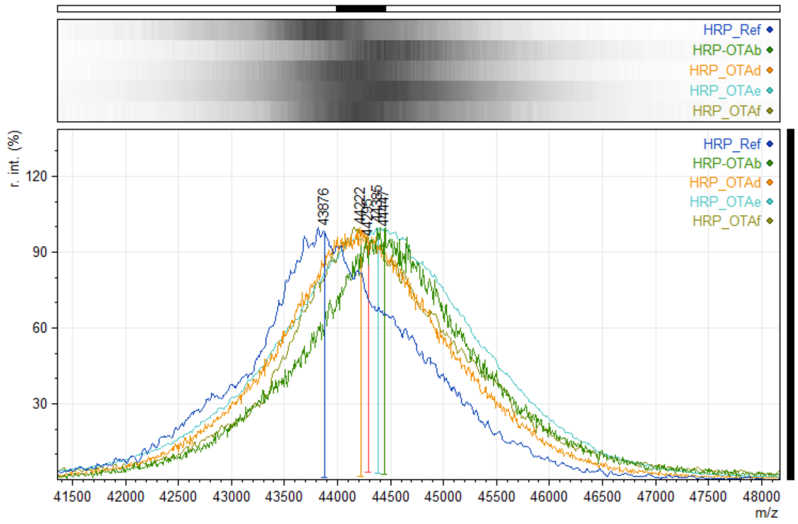

Figure S4. MALDI-TOF-MS spectra (singly charged ions) of HRP (blue) and the corresponding conjugates with haptens OTA*b* (green), OTA*d* (orange), OTA*e* (cyan), and OTA*f* (olive).

8. Antibody generation

Two 2-kg female New Zealand white rabbits were immunized with 21-day intervals by subcutaneous injection of 0.3 mg of BSA–hapten conjugate in 1 mL of a 1:1 emulsion between sterile 100 mM phosphate, pH 7.4, and Freund's adjuvant (complete for the first dose and incomplete for subsequent boosts). Ten days after the fourth injection, rabbits were exsanguinated by intracardiac puncture. Blood samples were allowed to coagulate overnight at 4 °C, and sera were separated by centrifugation (3000×g, 20 min). Finally, antibodies were precipitated with 1 volume of saturated ammonium sulfate solution. Salting out was performed twice, and precipitates were stored at 4 °C.

Four two-month old female Balb/c mice were immunized by intraperitoneal injection of 0.1 mg of BSA–OTA*b* or BSA–OTA*d* conjugate in 200 µL of a water-in-oil emulsion that was prepared using Freund's adjuvants as described above for rabbit immunization. Three doses were applied with 21-day intervals, and a fourth injection in sterile PBS with the same amount of protein conjugate was given at least 21 days after the third injection and 4 days before the animal was sacrificed. Hybridomas were prepared by fusion of mouse myeloma cells with lymphocytes b from two equally-immunized mice.

Antibody-producing cells were screened by a double sequential procedure. Twelve days after cell fusion, hybridoma culture supernatants were first screened by differential competitive ELISA on microtiter plates coated with 0.1 µg/mL (100 µL per well) of the homologous OVA–hapten conjugate. Fifty microliter of each supernatant was added to two adjacent wells of an ELISA plate, one containing 50 µL of PBS (blank) and the other containing 50 µL of 200 nM OTA in PBS. The ratio between the signals of both wells was used as the criterion for selecting the antibodies with the highest affinity. Fresh culture medium was added to those culture plate wells giving signals higher than 3.0 in the absence of mycotoxin. Next day, they were reevaluated by checkerboard competitive ELISA. This second screening assay was carried out with two coating concentrations of the homologous OVA–hapten conjugate (0.01 and 0.1 µg/mL), four supernatant dilutions (1/10, 1/50, 1/250, and 1/1250), and three OTA levels (0, 10, and 100 nM). Hybridomas selected from the first and the second screening assays were cloned twice by limiting dilution and cryopreserved in liquid nitrogen. Monoclonal antibodies were purified from late stationary phase hybridoma cell cultures by affinity chromatography using 5-mL protein G columns from GE Healthcare (Uppsala, Sweden) and stored at 4 °C as ammonium sulfate precipitates.
